# Species-level evaluation of the human respiratory microbiome

**DOI:** 10.1093/gigascience/giaa038

**Published:** 2020-04-16

**Authors:** Olufunmilola Ibironke, Lora R McGuinness, Shou-En Lu, Yaquan Wang, Sabiha Hussain, Clifford P Weisel, Lee J Kerkhof

**Affiliations:** 1 Environmental and Occupational Health Sciences Institute, School of Public Health, Rutgers–the State University of New Jersey, 170 Frelinghuysen Road, Piscataway, NJ, USA 08854, NJ, USA; 2 Department of Marine and Coastal Sciences, Rutgers–the State University of New Jersey, 71 Dudley Road, New Brunswick, NJ USA 08901; 3 Department of Pulmonary Medicine, Rutgers University-Robert Wood Johnsonchool, 125 Paterson Street, Suite 5200B New Brunswick, NJ 08901

**Keywords:** human respiratory microbiome, rRNA operon profiling, bacterial species identification, MinION, lung-enriched bacterial species

## Abstract

**Background:**

Changes to human respiratory tract microbiome may contribute significantly to the progression of respiratory diseases. However, there are few studies examining the relative abundance of microbial communities at the species level along the human respiratory tract.

**Findings:**

Bronchoalveolar lavage, throat swab, mouth rinse, and nasal swab samples were collected from 5 participants. Bacterial ribosomal operons were sequenced using the Oxford Nanopore MinION to determine the relative abundance of bacterial species in 4 compartments along the respiratory tract. More than 1.8 million raw operon reads were obtained from the participants with ∼600,000 rRNA reads passing quality assurance/quality control (70–95% identify; >1,200 bp alignment) by Discontiguous MegaBLAST against the EZ BioCloud 16S rRNA gene database. Nearly 3,600 bacterial species were detected overall (>750 bacterial species within the 5 dominant phyla: Firmicutes, Proteobacteria, Actinobacteria, Bacteroidetes, and Fusobacteria. The relative abundance of bacterial species along the respiratory tract indicated that most microbes (95%) were being passively transported from outside into the lung. However, a small percentage (<5%) of bacterial species were at higher abundance within the lavage samples. The most abundant lung-enriched bacterial species were *Veillonella dispar* and *Veillonella atypica* while the most abundant mouth-associated bacterial species were *Streptococcus infantis* and *Streptococcus mitis*.

**Conclusions:**

Most bacteria detected in lower respiratory samples do not seem to colonize the lung. However, >100 bacterial species were found to be enriched in bronchoalveolar lavage samples (compared to mouth/nose) and may play a substantial role in lung health.

## Context

The microbiome of the human lung has been investigated via high-throughput, short-read molecular DNA technologies and found to contribute significantly to health and respiratory diseases [1–9]. Specifically, the lung microbiome has been associated with diseases such as cystic fibrosis [10–15], chronic obstructive pulmonary disease [16–18], and asthma [19–23]. In addition, there is increasing evidence that changes to the lung microbiome may contribute to the progression of lung diseases [24]. Other studies have examined the contribution of the microbiome from the upper respiratory tract to the bacterial community in bronchoalveolar lavage (BAL) samples from healthy individuals in order to assess the resident versus transient microbes of the lung [25–28].

Prior studies have proposed and supported an “adapted island model,” suggesting that microbial communities within healthy lungs are changed by the interplay of immigration and elimination of bacterial species [4, 29–31]. For example, Venkataraman et al. [32] used a neutral community model to determine the proportion of microbial DNA originating from lung-adapted bacteria compared to those dispersed to the lung from other body sites. The study concluded that the neutral distribution of microbes dispersed from the mouth is consistent with the composition of the healthy lung microbiome [32]. Another group investigated the contribution of mouth and nose as source for bacterial communities for the lung (and gut) and reported that microbes are predominantly shared between mouth and lung while the nose microbiome contributes little to the lung microbiome in healthy individuals [33]. Unfortunately, most of these studies sampled only 2 locations to determine the microbial community changes along the respiratory tract. This approach would then be highly dependent on discerning differences within the end member samples without the possibility of verification. Furthermore, many studies used short variable regions of the 16S ribosomal RNA (rRNA) gene to analyze the respiratory tract microbiome. This short-read approach often resolves only at the bacterial genus to phylum levels. Therefore, changes in relative abundance for different bacterial species or strain levels along the respiratory tract would remain obscured.

**Figure 1:**
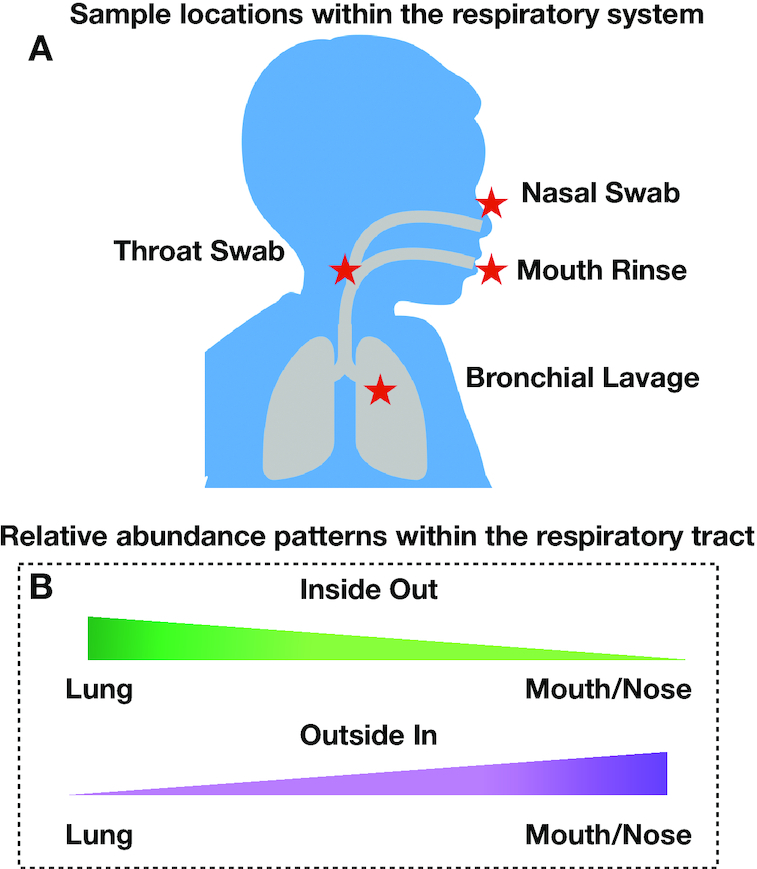
Location of respiratory samples collected in the present study (A) and conceptual model of relative abundance patterns within the respiratory tract (B).

In this study we used the Oxford Nanopore MinION to sequence nearly complete bacterial ribosomal operons, resulting in longer sequencing reads [34, 35] with species-level detection [36–38] in respiratory tract samples from 5-6 participants. We chose MinION rRNA operon profiling because it has been shown to accurately discern operational taxonomic units (OTUs) at the species level for reads with ≥79% identity, does not generate detectable chimeras, and provides a quantitative response for the top 100 numerically abundant OTUs [39]. Our hypothesis was that microbial populations living within the lung will display a relative abundance gradient along the respiratory tract. Therefore, samples were collected by BAL (indicated as “lung” or "LAV in the figures), throat swab, mouth wash, and nasal swab for rRNA operon profiling (Fig. 1A). Our hope was to distinguish those bacteria that displayed an outside-in pattern (highest relative abundance in mouth/nose) from those bacteria with an inside-out distribution (highest relative abundance in the lung compared with the mouth/nose) (Fig. 1B). The critical sample to assess this pattern is the throat swab, representing an intermediate relative abundance compared with the end-member samples. Our efforts identified a small subset of bacteria in the respiratory tract that conform to the inside-out model, potentially colonizing the lower respiratory tract after introduction from the outside. Understanding which specific bacteria can inhabit the lower respiratory tract has implications for assessing both opportunistic infections and which microbiota constitute a “healthy lung microbiota” for the development of lung-related diseases.

### Data description

Raw MinION sequence reads were collected as fast5 files with MinKnow (Oxford Nanopore Technologies), basecalled, separated by barcode, and converted to fastq files using Albacore (v 2.2.7). Reads between 3,700 and 5,700 bp in length from each sample were imported into Geneious (v 11) and screened against the EZ BioCloud 16S rRNA gene database [39] by Discontinuous MegaBLAST to determine OTUs [38]. The top hit data were exported as a .csv file and analyzed using pivot tables in Excel. Fastq data are available at NCBI SRA (Bioproject No. PRJNA564314).

## Methods

### Study approval

This study was approved by the Institutional Review Board of Rutgers, The State University of New Brunswick (protocol No. 20,140,000,953). All study participants provided signed written informed consent prior to any study interactions.

### Human subjects for the study

Six adult volunteers were recruited from patients who presented at Robert Wood Johnson Hospital for a scheduled diagnostic lavage primarily owing to a suspicious shadow on a lung x-ray. They were asked by the admitting clinician (S.H.) whether they were interested in participating in a research study in which excess lavage sample would be analyzed for bacteria in their lung and they would provide a series of non-invasive samples (e.g., throat and nose swab, oral cavity rinse). They were assured that their choice of whether to participate would not affect their medical care. The follow-up diagnosis was not obtained for these participants.

### Bacterial DNA extractions and purification

BAL, throat swab, oral cavity rinse, and nasal swab collection was performed or overseen by the attending physician (S.H.). DNA from all samples was purified using a direct, phenol/chloroform extraction for microbial community analysis [40] and stored at −80°C until used for PCR analysis.

### rRNA operon amplification

Near full-length bacterial operons were amplified with the 16S rRNA-27Forward primer (5′ AGA GTT TGA TCC TGG CTC AG 3′) [41] and the 23S rRNA-2241Reverse primer (5′ ACC GCC CCA GTH AAA CT 3′) [42], 2 µL of BAL (<1 ng template DNA), throat swab (<1 ng template DNA), nasal swab (<1 ng template DNA), and oral cavity rinse extract (<10 ng template DNA), and a Hi-Fidelity Taq polymerase (Bimake LLC, Houston, TX, USA) as previously described [38]. Ribosomal operons were amplified via touchdown PCR: initial denaturation was 5 min at 95°C; 2 cycles of 95°C/20 sec for denaturation, 68°C/15 sec for primer annealing, 72°C/75 sec for extension; then 2 cycles of 66°C for primer annealing; 2 cycles of 64°C for primer annealing; 2 cycles of 62°C for primer annealing—all with denaturation/extension; followed by 22 cycles of denaturation, 60°C/15 sec for primer annealing, 72°C/90 sec for extension; and a final extension at 72°C for 5 min. At the end of the 16th cycle (8 touchdown + 8 standard cycles), 12 µL of amplification mixture was removed and stored at −80°C. The amplification was allowed to proceed until 30 cycles were completed and the PCR product was visualized by means of agarose gel electrophoresis. Following verification of successful amplification by agarose gel electrophoresis, the 16-cycle PCR products were purified by AMPure bead clean-up as described above and a barcode amplification was performed. Barcode amplification conditions were 5 min at 95°C, followed by 30 cycles of 95°C for 20 sec, 60°C for 15 sec, and 72°C for 1:15 sec, followed by extension cycle at 72°C for 5 min. Barcoded rRNA amplicons were visualized and quantified by agarose gel electrophoresis.

### Library preparation and sequencing by MinION

MinION library construction used the 1D sequencing kit (SQK-LSK108-Oxford Nanopore; Oxford, UK). Two 12-barcoded amplicons (1,800 ng total in each) were combined, end-repaired, and dA-tailed in accordance with ONT instructions using NEB kits (New England Biolabs, Ipswich, MA, USA) and the modified Ampure bead purification described above. Ligation of the ONT adaptor used the Blunt/TA ligase master mix (NEB) with an addition of 1 µL of freshly prepared ATP solution (∼4 mg/mL) to facilitate ligation. All libraries were analyzed on R9.4 flow cells. To determine the contribution of rRNA operon sequences from PCR reagents and ONT sequencing kits, it was necessary to sequence the PCR negatives from our amplifications. Unfortunately, the original PCR negative results were accidently discarded and the LSK 108 kit that we used for these particular studies is no longer commercially available. Therefore, to address this “kitome” issue, we returned to the original DNA from Subject 15 (BAL, throat, mouth, and nose), re-amplified as described in the Methods section, and performed a sequencing reaction on both PCR-negative and PCR-positive samples with the LSK 109 kit. Analysis of this “kitome” indicated a 4,000-fold difference in sequence read numbers passing QA/QC (13 negative reads vs 41,135 positive reads), representing a possible contamination of 0.03% (Supplementary Fig. S5).

### Quality control

BAL, throat swab, oral cavity rinse, and nasal swab samples were collected from 6 participants, DNA was extracted, and rRNA operons were amplified (with universal rRNA operon primers and barcode primers). Unfortunately, 1 lavage sample from Subject 1 failed to properly amplify (Supplementary Fig. S1) and the remaining respiratory samples from this participant were included in overall community analysis but the samples from this particular participant were not characterized for lung enrichment by relative abundance. A total of ∼1.8 × 10^6^ raw reads were obtained, of which ∼1.2 × 10^6^ reads passed Albacore basecalling and were separated by barcode. After size selection (3.7–5.7 kb), a total of 623,271 barcoded sequences were screened against the EZ Biocloud database by Discontinuous MegaBLAST (Supplementary Fig. S2). Of these BLASTED reads, a total of 599,053 sequences passed an additional QA/QC step, having an identity between 70% and 95% and an alignment with >1,200 bp of the 16S rRNA genes in the database (Supplementary Fig. S3).

### Data validation

**Figure 2:**
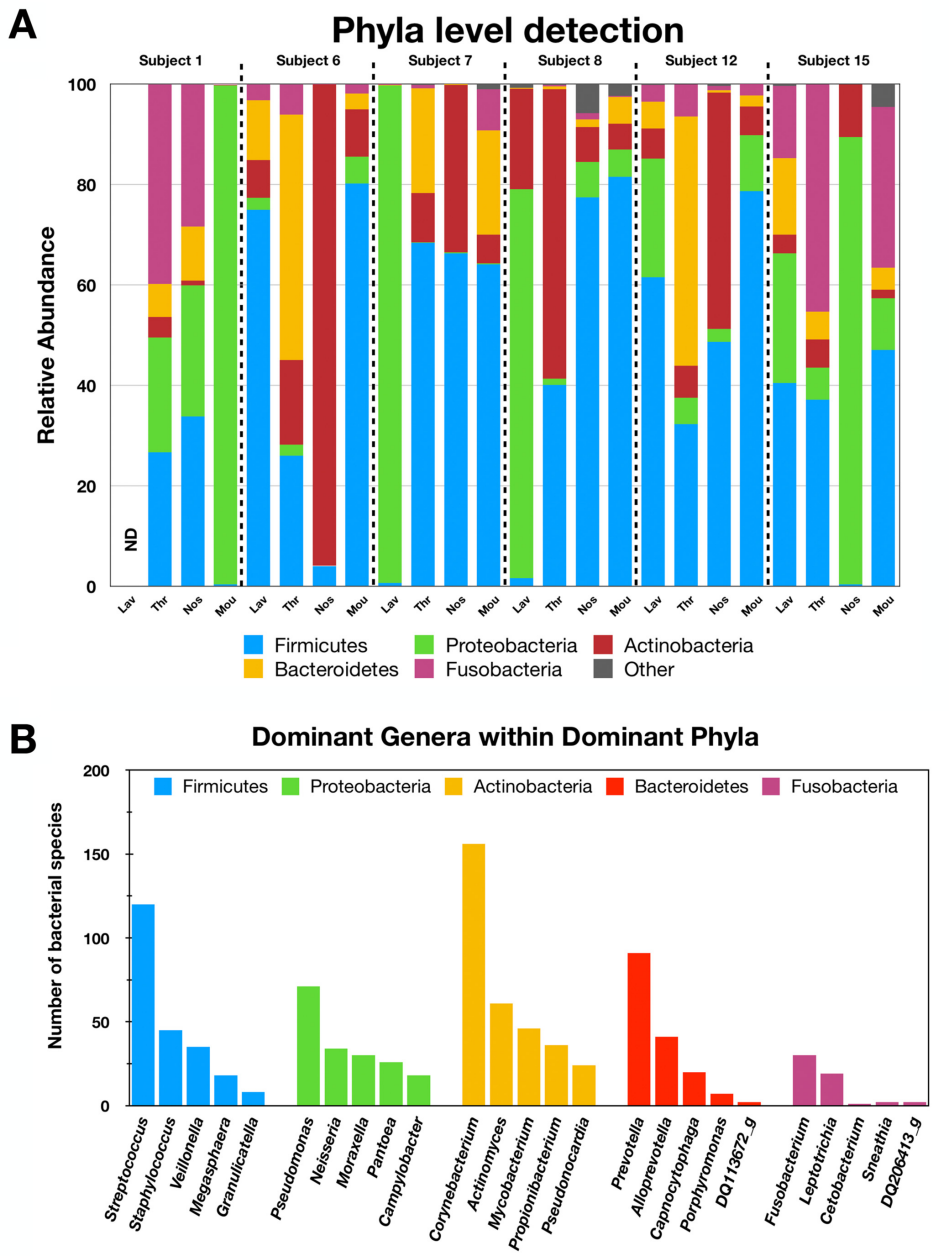
Relative abundance of bacterial phyla within respiratory samples for the various participants as indicated (A) and the number of bacterial species with the dominant genera/phyla across all participants (B).

**Figure 3:**
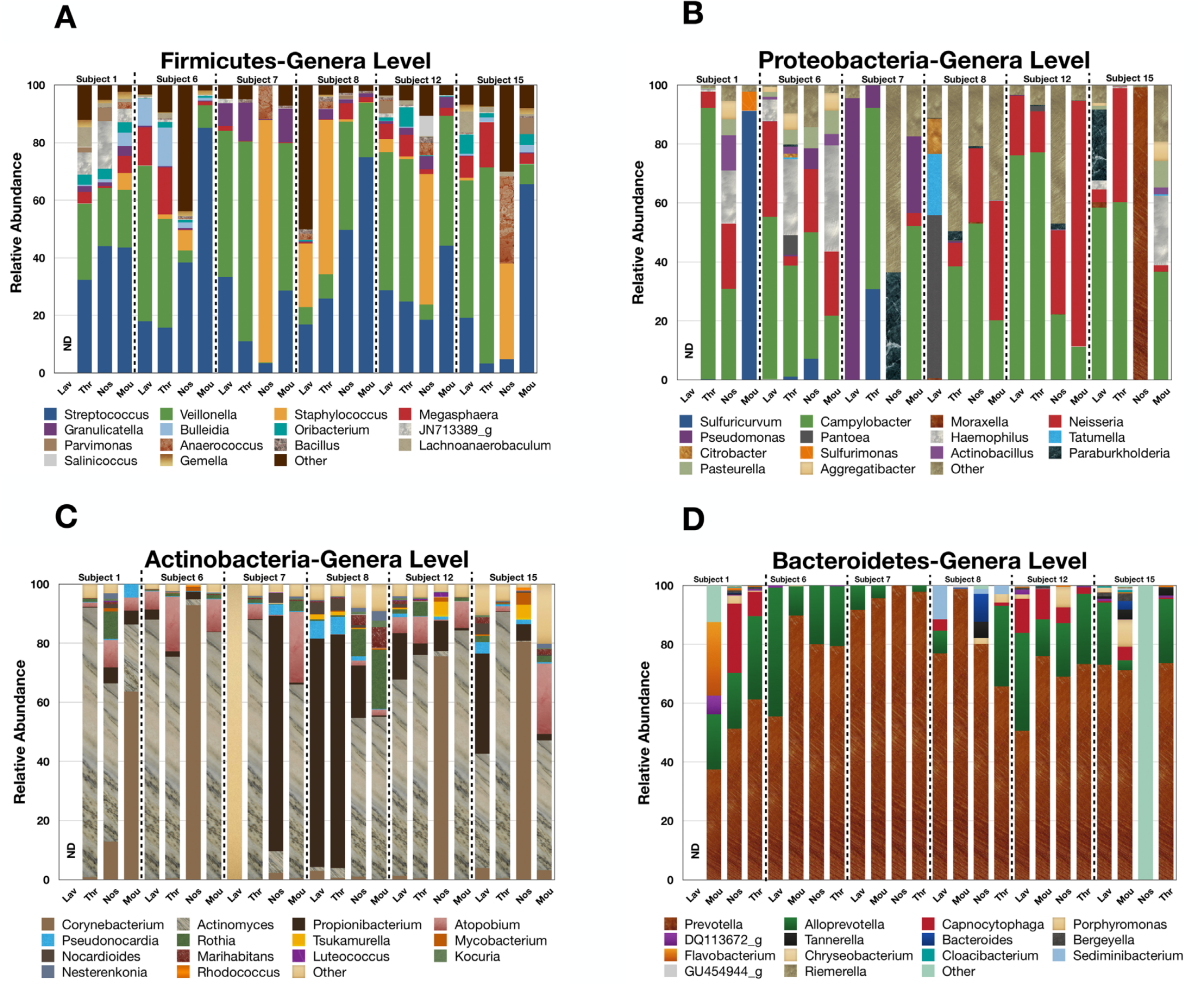
Relative abundance of the top 15 genera within the dominant phyla across all participants as indicated.

The BLAST screening indicated that the respiratory tract was dominated by 5 phyla: Firmicutes, Proteobacteria, Actinobacteria, Bacteroidetes, and Fusobacteria (representing >98% of the QA/QC reads) (Fig. 2A). The number of different species within the top 5 genera of these abundant phyla are presented in Fig. 2B while the relative abundances of the 15 most abundant genera within the dominant phyla are presented in Fig. 3. The relative abundance data indicate that the Firmicutes are mostly *Streptococcus* and *Veillonella* genera in lavage samples for the various participants. The Protobacteria are largely *Campylobacter* and *Neisseria* genera, with the exception of the lavage samples from Subject 7 (*Pseudomonas*) and Subject 8 (*Pantoea*). The Actinobacteria are mainly *Actinomyces* in Subjects 6, 12, and 15 and *Propionibacterium* or other bacteria in lavage samaples from Subjects 7 and 8, while the Bacteroidetes were dominated by *Prevotella* genera. Overall, the rRNA operon profiling detected ∼3,600 bacterial species with >750 species present within the dominant phyla. The most abundant bacterial species across all respiratory tract samples were *Veillonella dispar, Streptococcus parasanguinis, Streptococcus infantis, Streptococcus mitis*, and *Veillonella atypica*. Interestingly, the lavage profiles from Subjects 7 and 8 were markedly different than those from Subjects 6, 12, and 15 for the Proteobacteria and the Actinobacteria, suggesting that these participants were experiencing a lung infection at the time of sampling.

To assess whether the overall lung microbiome differed from throat, nose, or mouth microbiome, the data were initially subjected to principal component analysis (PCA) based on a Bray-Curtis dissimilarity index. PCA included log counts data for family- and species-level resolutions. There was no clear separation between lung and throat, nose, or mouth (PC1, 18%; PC2, 12% for species-level resolution) (Supplementary Fig. S4), indicating that any differences between the microbial communities are minor. The throat microbiome, compared with nose and mouth, was found to be the most similar to lung microbiome, with Bray-Curtis dissimilarity index averaging 0.68 ± 0.21 (species-level resolution) and 0.66 ± 0.23 (family-level resolution) across all participants. We also examined whether the samples from the different respiratory tract compartments differed for the individual participants. Similarly, there was no clear separation of bacterial community at the different compartments between the participants (PC1, 26%; PC2, 14% for species-level resolution; data not shown).

**Figure 4:**
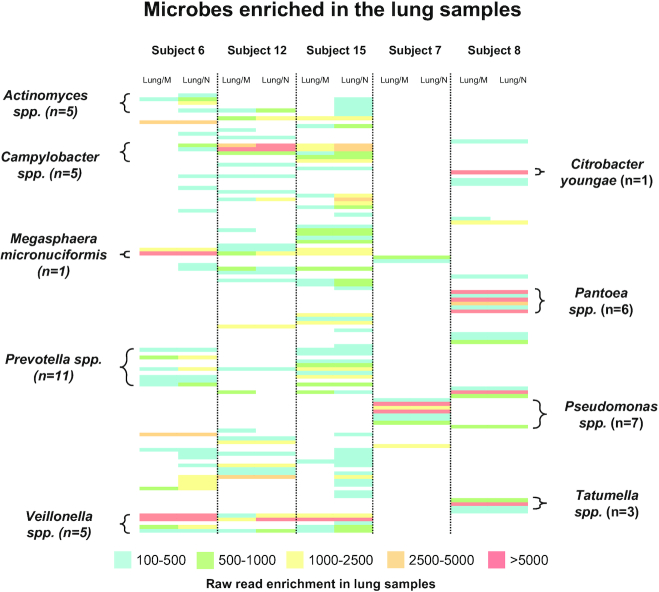
Heat map of lung-enriched bacterial species (i.e., lung reads vs mouth reads or lung reads vs nose reads as indicated) for the various participants. The number of bacterial species within specific genera are indicated. Full description of lung-enriched taxa is presented in Supplemental Table 1.

To identify lung-enriched bacteria genera and species, we subtracted the read counts of mouth and nose from BAL counts after normalization for each participant. More than 1,300 lung-enriched bacterial species were discerned across all samples. However, most of these differences in read counts were <50, which may represent methodological variation in raw read results from MinION sequencing. Our prior work has shown that replicate read numbers of >100 have a coefficient of variation of ∼12% or less [39]. Therefore, a conservative threshold of 150 read differences was used to define those bacteria enriched in the lower respiratory tract. This yielded 114 bacterial species from all participants with a stronger rRNA operon signal in BAL samples compared with the higher respiratory tract samples (Supplementary Table S1). To determine whether comparable lung enrichment was observed for the participants for particular OTUs, a heat map was generated using the lung-mouth and lung-nose read differences in relative abundances that were >150 reads (Fig. 4). Overall, those lung-enriched OTUs were nearly equally in the BAL samples for Subjects 6, 12, and 15. The predominant lung-enriched bacterial genera for this group were *Veillonella spp*., *Prevotella spp*., *Campylobacter spp*., *Actinomyces spp*., and *Megasphaera micronuciformis*. In contrast, Subjects 7 and 8 were largely missing these particular OTUs and were enriched in *Tatumella spp*., *Pseudomonas spp*., *Pantoea spp*, and *Citrobacter youngae*, consistent with a lung infection at the time of sampling. For many of these genera within bronchial lavage, 3–11 different bacterial species were detected. Interestingly, we did not detect any lung-enriched bacterial species that were present in all participants. In addition, almost all bacteria species detected in the lung samples are also detected in the throat samples.

**Figure 5:**
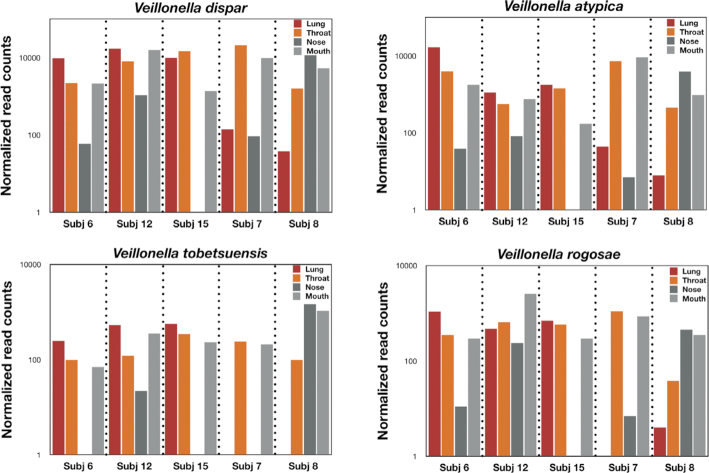
Histogram of normalized reads for the respiratory compartments of the different participants for *Veillonella* spp. as indicated.

**Figure 6:**
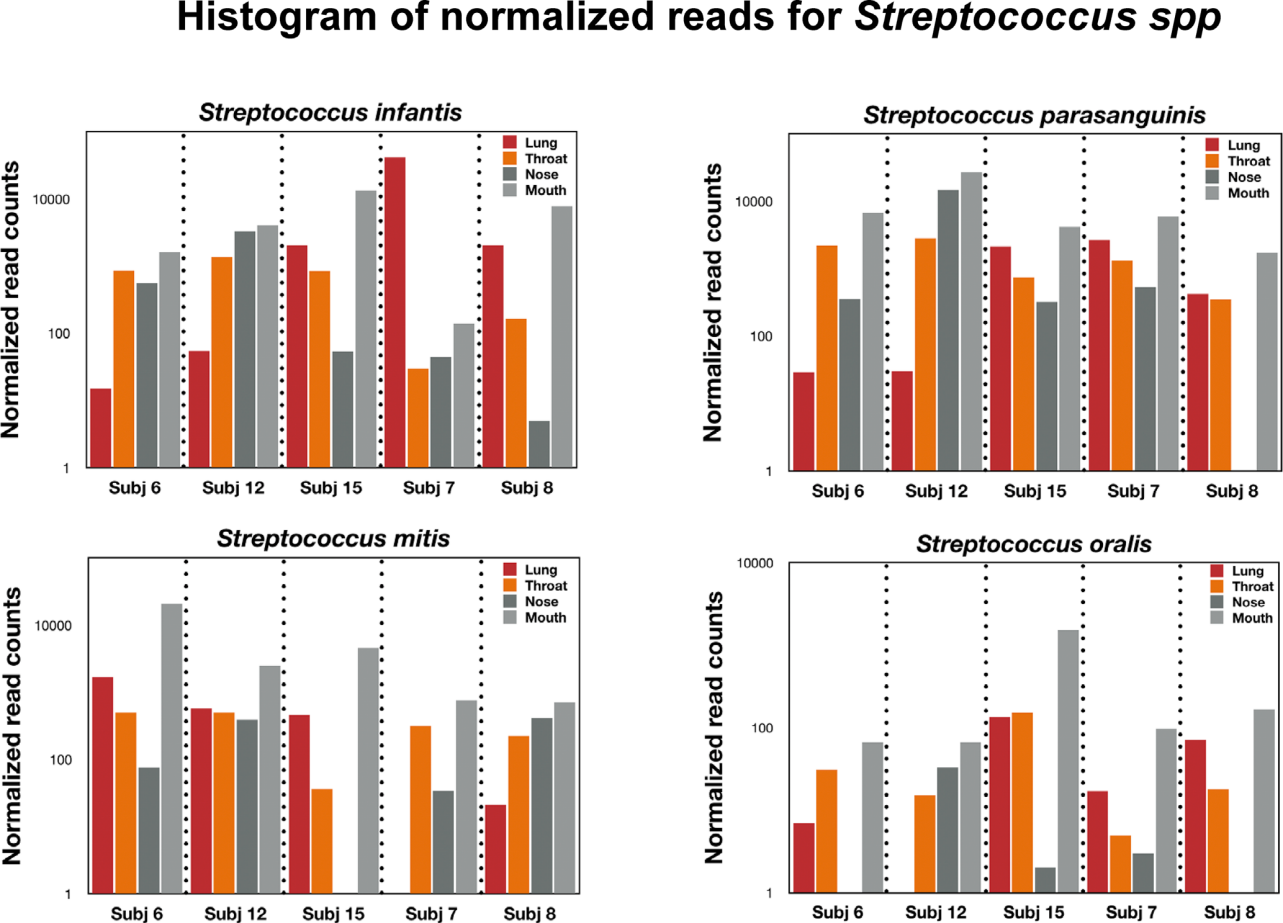
Histogram of normalized reads for the respiratory compartments of the different participants for *Streptococcus* spp. as indicated.

To verify that we can detect bacterial species that are in higher abundance in the lung, we compared reads across all 4 respiratory compartments. For *Veillonella spp*. (the most abundant lung-enriched species in Subjects 6, 12, and 15), *Veillonella dispar, Veillonella atypica, Veillonella tobetsuensis, and Veillonella rogosae* generally demonstrated a higher relative abundance in lung samples compared with mouth and nose samples while the throat swab represented an intermediate relative abundance (Fig. 5). For Subjects 7 and 8, a different pattern was observed: the *Veillonella* reads for the lung were suppressed or absent. For example, *V. tobetsuensis* was not detected in lung samples from either Subject 7 or 8, while *V. rogosae* was absent from Subject 7 and in very low abundance for Subject 8. Interestingly, the throat/mouth/nose samples for *Veillonella* spp. in these participants all had higher abundances than the lung samples. Conversely, those bacterial species that yielded a negative number when upper respiratory samples were subtracted from bronchial lavage samples (mouth/nose enriched) also displayed an intermediate signal for throat samples for Subjects 6, 12, and 15 (Fig. 6). For example, the relative abundance for *S. infantis*, *S. parasanguinis*, and *Streptococcus oralis* generally displayed an outside-in pattern for Subjects 6, 12, and 15, while Subject 7 displayed higher abundances in the lung for *S. infantis*, consistent with a lung infection.

## Discussion

DNA-based microbial analysis has identified changes in the human respiratory microbiome for many lung diseases [10–23]. Most of these earlier studies used 2 end-member sites (e.g., lung and mouth) to characterize the respiratory microbiome. For example, lung bacterial communities were found in lower abundance compared to the upper respiratory tract [25] and differences between lung and upper respiratory bacterial communities have been described at the genus: family: phylum level [29, 33, 43]. However, because of the low biomass within the lung, end-member analysis to determine the microbial differences along the respiratory tract is difficult to verify. Furthermore, studies resolving only from the bacterial genus to phylum levels will not detect differences within bacterial species or strain levels from the lung. In the present study, near full-length rRNA operon sequence reads were used to discern those bacteria capable of colonizing the lung from those being passively transported and eliminated by processes that clear the respiratory tract. Our long-read approach allowed for both species-level detection of bacteria and the assessment of relative abundances along the respiratory tract to distinguish bacterial species enriched in lung samples. Additionally, the inclusion of throat samples represents an intermediate location that enabled verification of relative changes in microbiome communities along the respiratory tract. The results demonstrate that <5% of bacterial species detected in the respiratory tract were enriched in the lung.

It is thought that in healthy individuals, the lung microbiome generally becomes inoculated by bacteria from the mouth and the community is maintained by the balance between immigration, colonization, and elimination processes [26]. In contrast, this balance in the “healthy” lung microbiome becomes disturbed during lung infection and diseases [3]. In the present study, we can observe a comparable displacement of the lung-enriched microbiome observed in Subjects 6, 12, and 15 by the lung-enriched community in Subjects 7 and 8. Specifically, high relative abundances of *Pseudomonas spp*. in Subject 7 and *Tatumella*and*Pantoea spp*. in Subject 8 were accompanied by a decrease in the relative abundances of *Actinomyces*, *Campylobacter*, *Prevotella*, and *Veillonella* species within their lungs. Our findings are consistent with other studies, which have implicated *Pseudomonas aeruginosa, Tatumella ptyseos*,and other Proteobacteria in chronic lung diseases [19, 44, 45], cystic fibrosis [46], or asthma [47]. Likewise, our findings are in agreement with prior work, which identified *Veillonella* spp. as one of the most abundant bacteria in the respiratory tract of healthy individuals [48] or *Prevotella spp*. as prevalent commensal colonizers of mucosal surfaces [49] and members of the “healthy” lung microbiome [19, 25]. Finally, an important caveat of this study is that our samples were collected at a single time point to distinguish those bacteria displaying a change in relative abundance along the respiratory tract. It would be helpful for future studies to sample the various respiratory compartments over time to delineate changes in the microbiome before, during, and after lung infections to monitor lung microbiome dynamics.9

### Re-use potential

Our study found >100 different bacterial species that are capable of colonizing the human lung and followed an inside-out distribution with respect to upper respiratory samples. Understanding which specific bacteria can colonize the lower respiratory tract will help in discerning which microbiota constitute a “healthy lung microbiota” and provide a diagnostic tool for studying the role of the microbiome in the development of lung-related diseases.

## Availability of Supporting Data and Materials

All raw sequence data are currently being made available at NCBI SRA (Bioproject No. PRJNA564314). Further supporting data are available in the *GigaScience* repository, GigaDB [50].

## Additional Files

**Supplemental Figure S1:** Agarose gel showing amplification of rRNA operons from Subjects 1, 6, and 12 as indicated.

**Supplemental Figure S2:** Summary data of read numbers for all participants using the MinION platform.

**Supplemental Figure S3:** Plot of percent identity vs alignment length for MinION raw reads against the EZ BioCloud database using Discontinuous MegaBLAST.

**Supplemental Figure S4:** PCA plot of samples located in different compartments from the various participants based on Bray-Curtis dissimilarity.

**Supplemental Figure S5:** Kitome analysis: agarose gel showing amplification of rRNA operons from Subject 15 (PCR negatives/positives as indicated) (A) and histogram of reads passing QA/QC for PCR negatives/positives (B).

**Supplemental Table S1:** Heat map of lung-enriched taxa.

## Abbreviations

ATP: adenosine triphosphate; BAL: bronchoalveolar lavage; BLAST: Basic Local Alignment Search Tool; bp: base pairs; NCBI: National Center for Biotechnology Information; ONT: Oxford Nanopore Technologies; OTU: operational taxonomic unit; PCA: principal component analysis; QA/QC: quality assurance/quality control; rRNA: ribosomal RNA; SRA: Sequence Read Archive.

## Consent for Publication

All authors of the manuscript have read and agreed to its content and are accountable for the accuracy and integrity of the manuscript.

## Competing Interests

The authors declare that they have no competing interests.

## Funding

This research was funded in part by an NIEHS Training Grant in Exposure Science 1T32ES019854 to C.P.W., a Rutgers University Center for Environmental Exposure and Disease (CEED) Pilot Project Grant (No. 5P30ES005022) to C.P.W. and L.J.K., and by Rutgers University Indirect Cost Return to L.J.K.

## Authors’ contributions

C.P.W, S.H., L.R.M., and L.J.K. conceived and designed the experiments. S.H. and associated post-doctoral students collected the respiratory samples. L.J.K. performed DNA extractions. O.I. amplified the rRNA operons and created the sequence libraries with L.J.K. L.J.K. performed the sequencing and developed the data analysis approach with L.R.M. and O.I. S.L. and Y.W. performed the principal component analysis. O.I., L.J.K., L.R.M., and C.P.W. discussed the findings and interpreted the results. O.I. and L.J.K. wrote the first draft. All authors read, edited, and approved the final manuscript.

## Supplementary Material

giaa038_GIGA-D-19-00352_Original_Submission

giaa038_GIGA-D-19-00352_Revision_1

giaa038_Response-to-Reviewer_Comments_Original_Submission.pdf

giaa038_Reviewer_1_Report_Original_Submission Mohammad Bahram -- 11/19/2019 Reviewed

giaa038_Reviewer_2_Report_Original_Submission Keith Robison -- 12/9/2019 Reviewed

giaa038_Reviewer_2_Report_Revision_1 Keith Robison -- 3/12/2020 Reviewed

giaa038_Supplemental_Files
