# Supplementary material for: Species-level evaluation of the human respiratory microbiome
Source: Gigascience. 2020 Apr 16;9(4):giaa038. doi: 10.1093/gigascience/giaa038 (PMC7162353; doi:10.1093/gigascience/giaa038)
Supplement: giaa038_GIGA-D-19-00352_Revision_1 [file giaa038_giga-d-19-00352_revision_1.pdf]

GigaScience

Species-Level Evaluation of the Human Respiratory Microbiome

--Manuscript Draft--

**Manuscript Number:**	GIGA-D-19-00352R1
**Full Title:**	Species-Level Evaluation of the Human Respiratory Microbiome
**Article Type:**	Data Note
**Funding Information:**	National Institute of Environmental Health Sciences (1T32ES019854) — Prof. Clifford P. Weisel
**Abstract:**	Background<br><br>Changes to human respiratory tract microbiome may contribute significantly to the progression of respiratory diseases. However, there are few studies examining the relative abundance of microbial communities at the species level along the human respiratory tract.<br><br>Findings<br><br>Bronchoalveolar lavage (BAL), throat swab, mouth rinse, and nasal swab samples were collected from 5 subjects. Bacterial ribosomal operons were sequenced using the Oxford Nanopore MinION to determine the relative abundance of bacterial species in 4 compartments along the respiratory tract. Over 1.8 million raw operon reads were obtained from the subjects with ~600K rRNA reads passing QA/QC (70-95% identify; >1200 bp alignment) by Discontinuous MegaBlast against the EZ BioCloud 16S rRNA gene database. Nearly 3600 bacterial species were detected overall (> 750 bacterial species within the 5 dominant phyla: Firmucutes, Proteobacteria, Actinobacteria, Bacteroidetes, and Fusobacteria . The relative abundance of bacterial species along the respiratory tract indicated most microbes (95%) were being passively transported from outside into the lung. However, a small percentage (<5%) of bacterial species were at higher abundance within the lavage samples. The most abundant lung-enriched bacterial species were Veillonella dispar and Veillonella atypica while the most abundant mouth-associated bacterial species were Streptococcus infantis and Streptococcus mitis .<br><br>Conclusions<br><br>Most bacteria detected in lower respiratory samples do not seem to colonize the lung. However, over 100 bacterial species were found to be enriched in bronchial lavage samples (compared to mouth/nose) and may play a significant role in lung health.
**Corresponding Author:**	Lee Kerkhof<br><br>UNITED STATES
**Corresponding Author Secondary Information:**	
**Corresponding Author's Institution:**	
**Corresponding Author's Secondary Institution:**	
**First Author:**	Lee Kerkhof
**First Author Secondary Information:**	
**Order of Authors:**	Lee Kerkhof<br>Olufunmilola Ibironke<br>Lora R. McGuinness<br>Shou-En Lu

Powered by Editorial Manager® and ProduXion Manager® from Aries Systems Corporation

	Yaquan Wang
	Sabiha Hussain
	Clifford P. Weisel
**Order of Authors Secondary Information:**	
**Response to Reviewers:**	********<br>Please also see the cover letter which has the response to the reviewers with figures which can not be included in this text box.<br>********<br><br>Response to reviewers<br><br>Reviewer #1:<br><br>Could the authors discuss whether the differences in sampling devices used may explain some of the differences observed?<br><br>Samples were collected by either rinse or swab. We do not observe higher similiarity between throat and nose or lavage and mouth (i.e. collection method). Rather the clustering has more to do with location within the respiratory tract as we had hypothesized and shown in Supplemental Figure 4.<br><br>Could they also discuss whether there are limitations regarding the quality of Nanopore sequence are sufficient for specific-level identification?<br><br>In our prior publication, MinION rRNA operon profiling was shown to accurately discern OTUs at the species-level for reads >79% identity, does not generate detectable chimeras, and provides a quantitative response for the top 100, numerically-abundant OTUs (Kerkhof et al., 2017).<br><br>We have changed lines 81-84 to reflect this. For the record, Cusco et al., 2018 and Benitez-Paez et al., 2017 who have also demonstrated species-level identification using rRNA operons and the MinION platform and our cited in our submission.<br><br>L131 - L132 Could the authors provide references for these primers.<br><br>Fixed<br><br>L201 - L204 Please provide the range of these values, and it would be good if the authors could compare these values community null model structure.<br><br>There are no range in values for the Principal Components (PC1, PC2) beyond what is presented. We have now included the average and standard deviation for the Bray-Curtis similarity indices across all subjects. We are unsure what null model the reviewer is referring to. However,<br><br>Reviewer #2:<br>The flowcell type does not appear to be in the manuscript; the specific pore version should be included (e.g. R9.4.1) to make this unambiguous. Ideally it would be in both the Data description and M&M sections<br><br>We indicated in the original submission that R9 flow cells were used on line 160. We have updated this text to R9.4 flow cells as requested. R9.4.1 flow cells were not yet available at the time of this study.<br>The lack of any form of negative control is a serious flaw in the experimental design. At a minimum a no sample control should have been (done) to control for "kitome"<br><br>We agree. Unfortunately, the LSK 108 kit that we used for these particular studies are no longer commercially available. However, in order to address the “kitome” issue, we have returned to a subset of the original DNA from Subject 15, amplified as described in the methods section, and performed a sequencing reaction with the LSK 109 kit. Our amplifications are shown here. Both PCR Negatives and PCR Positives were sequenced.

Powered by Editorial Manager® and ProduXion Manager® from Aries Systems Corporation

During a 3 hr run, the PCR negatives generated 13 sequences which passed QA/QC while the PCR positives generated over 40,000 sequences which passed QA/QC. This represents a 4000-fold difference in read numbers between negative and positive samples and a possible contamination of 0.03% by the "kitome".

The informatics methodology used to separate the nanopore reads by barcode is not described. It would also be useful to describe how many reads were not assigned a barcode.

Albacore software basecalls and separates MinION reads by barcode as indicated by line 100 in our original submission.

Supplemental Fig 2. illustrates the number of raw reads, those basecalled/barcoded by Albacore, and those reads which pass QA/QC (3700> x >5700 bp).

My major concern is whether the accuracy of nanopore reads is sufficient to make species-level calls bases on rRNA alignments. For example, Veillonella dispar and Veillonella atypica are 98% identical for rRNA, whereas raw nanopore reads have accuracies ranging below 95%. Hence much of the species-level analysis may be artifactual. This is amply illustrated by Supplemental Figure 3.

We disagree with the reviewer's concern here. As cited above, we have demonstrated accurate OTU calling at the species-level for individual MinION ribosomal operon reads >79% identity screened against a 16S rRNA gene database, no detectable chimeras, and a quantitative capability for the top 100, numerically-abundant OTUs using the MinION in our prior publication (Kerkhof et al., 2017).

In our prior study, we did not determine "how low we could go" with respect to accurate OTU calling at the species level. Supplemental Fig. 3 has the 79% identity marked by a dashed line.

In this figure, is "Percent Similarity" really appropriate or is it "Percent Identity" -- are there any mismatches which are scored differently than other mismatches?

Fixed. All mismatches are scored the same.

There is also the concern that MegaBLAST may not be reliable for identifying a top matching sequence depending on parameter settings, a topic which has been discussed (e.g. Shah et al 2018). Tools such as VSEARCH have been explicitly designed for identifying the best match amongst closely related sequences.

We disagree. For the reviewer, we reproduce our in silico testing of 3 different 16S rRNA genes which have been mutated in silico to emulate MinION sequence data (79%-100% identity) from Kerkhof et al., 2017. The table reports the top hit (Description) and the MegaBLAST results. All Discontinuous MegaBlast searches using targets with introduced errors were accurately retrieved from the database at the species (and the strain) levels as shown below. This is possible because the MinION encompasses the entire 16S rRNA gene rather than a short variable region.

The shading and 3D effects for Supplemental Figure 2 are distracting and add nothing. This figure would be improved by removing them.

We disagree with the reviewer here. This particular figure has been presented on posters at a number of national meetings and the vast majority of people who view our poster comment on the graphics and like them very much.

Supplemental Figure 4 should use a better visual scheme for differentiating the datapoints; the use of soft colors is difficult to disambiguate for individuals with atypical color perception. Given that the four sample sites begin with different letters, incorporating these as markers would be ideal, as it would eliminate the need to remap sites to colors or shapes. The significance of the overlaid ovals should be described in the legend.

	We agree with the reviewer here and have changed the figure to black/white with different symbols for clarity. The ovals have been removed.
**Additional Information:**	
**Question**	**Response**
Are you submitting this manuscript to a special series or article collection?	No
**Experimental design and statistics**<br><br>Full details of the experimental design and statistical methods used should be given in the Methods section, as detailed in our Minimum Standards Reporting Checklist. Information essential to interpreting the data presented should be made available in the figure legends.<br><br>Have you included all the information requested in your manuscript?	Yes
**Resources**<br><br>A description of all resources used, including antibodies, cell lines, animals and software tools, with enough information to allow them to be uniquely identified, should be included in the Methods section. Authors are strongly encouraged to cite Research Resource Identifiers (RRIDs) for antibodies, model organisms and tools, where possible.<br><br>Have you included the information requested as detailed in our Minimum Standards Reporting Checklist?	Yes
**Availability of data and materials**<br><br>All datasets and code on which the conclusions of the paper rely must be either included in your submission or deposited in publicly available repositories (where available and ethically appropriate), referencing such data using a unique identifier in the references and in the “Availability of Data and Materials”	Yes

Powered by Editorial Manager® and ProduXion Manager® from Aries Systems Corporation

section of your manuscript.<br><br>Have you have met the above requirement as detailed in our Minimum Standards Reporting Checklist?	

Manuscript with DOI

Click here to access/download;Manuscript;Human_Respiratory_MInION_R1 w

Species-Level Evaluation of the Human Respiratory Microbiome

Olufunmilola Ibironke [1], Lora R. McGuinness [2], Shou-En Lu [1], Yaquan Wang [1], Sabiha Hussain [3], Clifford P. Weisel [1], and Lee J. Kerkhof [2]*

[1] Environmental and Occupational Health Sciences Institute, School of Public Health, Rutgers- the State University of New Jersey

[2] Department of Marine and Coastal Sciences, Rutgers- the State University of New Jersey

‘[3] Department of Pulmonary Medicine, Rutgers University Medical School

Authors email:

OI: oai5@gsbs.rutgers.edu

LRM: mcguinne@marine .rutgers.edu;

SL: sl1020@sph.rutgers.edu;

YW: yw505@sph.rutgers.edu;

SH: hussain.sabiha@gmail.com;

CPW: cpweisel@eohsi.rutgers.edu;

LJK: kerkhof@marine.rutgers.edu

*Corresponding Author: LJK

Abstract

Background: Changes to human respiratory tract microbiome may contribute significantly to the progression of respiratory diseases. However, there are few studies examining the relative abundance of microbial communities at the species level along the human respiratory tract.

Findings: Bronchoalveolar lavage (BAL), throat swab, mouth rinse, and nasal swab samples were collected from 5 subjects. Bacterial ribosomal operons were sequenced using the Oxford Nanopore MinION to determine the relative abundance of bacterial species in 4 compartments along the respiratory tract. Over 1.8 million raw operon reads were obtained from the subjects with ~600K rRNA reads passing QA/QC (70-95% identify; >1200 bp alignment) by Discontinuous MegaBlast against the EZ BioCloud 16S rRNA gene database. Nearly 3600 bacterial species were detected overall (> 750 bacterial species within the 5 dominant phyla: *Firmucutes, Proteobacteria, Actinobacteria, Bacteroidetes,* and *Fusobacteria*. The relative abundance of bacterial species along the respiratory tract indicated most microbes (95%) were being passively transported from outside into the lung. However, a small percentage (<5%) of bacterial species were at higher abundance within the lavage samples. The most abundant lung-enriched bacterial species were *Veillonella dispar* and *Veillonella atypica* while the most abundant mouth-associated bacterial species were *Streptococcus infantis* and *Streptococcus mitis*.

Conclusions: Most bacteria detected in lower respiratory samples do not seem to colonize the lung. However, over 100 bacterial species were found to be enriched in

bronchial lavage samples (compared to mouth/nose) and may play a significant role in lung health.

Keywords: Human respiratory microbiome, rRNA operon profiling, bacterial species identification, MinION, lung-enriched bacterial species.

Context

The microbiome of the human lung has been investigated via high-throughput, short-read molecular DNA technologies and found to contribute significantly to health and respiratory diseases [1-9]. Specifically, the lung microbiome has been associated with diseases such as cystic fibrosis [10-15], chronic obstructive pulmonary disease [16-18] and asthma [19-23]. Additionally, there is increasing evidence that changes to the lung microbiome may contribute to the progression of lung diseases [24]. Other studies have examined the contribution of the microbiome from the upper respiratory tract to the bacterial community in bronchial lavage from healthy individuals in order to assess the resident versus transient microbes of the lung [25-28].

Prior studies have proposed and supported an “adapted island model”, suggesting microbial communities within healthy lungs are changed by the interplay of immigration and elimination of bacterial species [4, 29-31]. For example, Venkataraman et al [32] employed a neutral community model to determine the proportion of microbial DNA originating from lung-adapted bacteria compared to those dispersed to the lung from other body sites. The study concluded that the neutral distribution of microbes

dispersed from the mouth is consistent with the composition of the healthy lung microbiome [32]. Another group investigated the contribution of mouth and nose as source for bacterial communities for the lung (and gut) and reported that microbes are predominantly shared between mouth and lung while the nose microbiome contributes little to the lung microbiome in healthy individuals [33]. Unfortunately, most of these studies sampled only 2 locations to determine the microbial community changes along the respiratory tract. This approach would then be highly dependent on discerning differences within the end member samples without the possibility of verification. Furthermore, many studies utilized short variable regions of the 16S rRNA gene to analyze the respiratory tract microbiome. This short-read approach often resolves only at the bacterial family to phylum levels. Therefore, changes in relative abundance for different bacterial species or strain levels along the respiratory tract would remain obscured.

In this study we utilized the Oxford Nanopore MinION to sequence nearly complete bacterial ribosomal operons, resulting in longer sequencing reads [34, 35] with species-level detection [36-38] in respiratory tract samples from 5 subjects. We chose MinION rRNA operon profiling since it has been shown to accurately discern OTUs at the species-level for reads $\geq$79% identity, does not generate detectable chimeras, and provides a quantitative response for the top 100, numerically-abundant OTUs [39]. Our hypothesis was that microbial populations living within the lung will display a relative abundance gradient along the respiratory tract. Therefore, samples were collected by bronchoalveolar lavage (BAL; indicated as “lung” in the figures), throat swab, mouth wash, and nasal swab for rRNA operon profiling (Fig 1A). Our hope was to distinguish

those bacteria which displayed an outside-in pattern (highest relative abundance in mouth/nose) from those bacteria with an inside-out distribution (highest relative abundance in the lung compared to the mouth/nose) (Fig 1B). The critical sample to assess this pattern is the throat swab, representing an intermediate relative abundance compared with the end-member samples. Our efforts identified a small subset of bacteria in the respiratory tract which conform to the inside-out model, potentially colonizing the lower respiratory tract after introduction from the outside. Understanding which specific bacteria can inhabit the lower respiratory tract has implications for assessing both opportunistic infections and which microbiota constitute a “healthy lung microbiota” for the development of lung related diseases.

Data description

Raw MinION sequence reads were collected as fast5 files with MinKnow (Oxford Nanopore Technologies), basecalled, separated by barcode, and converted to fastq files using Albacore (v 2.2.7). Reads between 3700-5700 bp in length from each sample were imported into Geneious (v 11) and screened against the EZ BioCloud 16S rRNA gene database [39] by Discontinuous MegaBLAST to determine operational taxonomic units (OTU) [38]. The top hit data were exported as a .csv file and analyzed using pivot tables in Excel. Fastq data is available at NCBI SRA (Bioproject # PRJNA564314).

Methods

Study Approval

This study was approved by the Institutional Review Board of Rutgers, The State University of New Brunswick (protocol #20140000953). All study subjects provided signed written informed consent prior to any study interactions.

Human Subjects for the study

Six adult volunteers were recruited from patients who presented at Robert Wood Johnson Hospital for a scheduled diagnostic lavage primarily due to a suspicious shadow on a lung x-ray. They were asked by the admitting clinician (SH) if they were interested in participating in a research study in which excess lavage sample will be analyzed for bacteria in their lung and provide a series of non-invasive samples (e.g. throat and nose swab, oral cavity rinse). They were assured that the answer as to whether they choice to participate would not affect their medical care. The follow-up diagnosis was not obtained for these subjects.

Bacterial DNA Extractions and Purification

Bronchial lavage (BAL), throat swabs, mouth wash, and nasal swabs collection was done or overseen by the attending physician (SH). DNA from BAL, throat swabs, mouth wash, and nasal swabs was purified using a using a direct, phenol/chloroform extraction for microbial community analysis [40] and stored at -80°C until used for PCR analysis.

rRNA Operon Amplification

Near full-length bacterial operons were amplified with the 16S rRNA-27Forward primer (5′ AGA GTT TGA TCC TGG CTC AG 3′) [41] and the 23S rRNA-2241Reverse primer (5′ ACC GCC CCA GTH AAA CT 3′) [42], 2 µL of BAL (<1 ng template DNA), throat swab (<1 ng template DNA), nasal swab (<1 ng template DNA), and mouth wash extract (<10 ng template DNA), and a Hi-Fidelity Taq polymerase (Biomake LLC, Houston, TX, USA) as previously described [38]. Ribosomal operons were amplified via touchdown PCR: Initial denaturation was 5 min at 95 ^{o}C; 2 cycles of 95 ^{o}C /20 secs for denaturation, 68 ^{o}C /15 secs for primer annealing, 72 ^{o}C / 75 secs for extension; then 2 cycles of 66 ^{o}C for primer annealing; 2 cycles of 64 ^{o}C for primer annealing; 2 cycles of 62 ^{o}C for primer annealing-all with denaturation/extension; followed by 22 cycles of denaturation, 60 ^{o}C /15 secs for primer annealing, 72 ^{0}C / 90 secs for extension; and a final extension at 72 ^{o}C for 5 min. At the end of the 16th cycle (8 touchdown + 8 standard cycles), 12 µl of amplification mixture was removed and stored at -80 ^{o}C. The amplification was allowed to proceed until 30 cycles was completed and the PCR product was visualized by agarose gel electrophoresis. Following verification of successful amplification by agarose gel electrophoresis, the 16 cycle PCR products were purified by AMPure bead clean-up as described above and a barcode amplification using the ONT barcoding kit was performed. Barcode amplification conditions were 5 min at 95 ^{o}C, followed by 30 cycles of 95 ^{o}C for 20 sec, 60 ^{o}C for 15 sec and 72 ^{o}C for 1:15 sec, followed by extension cycle at 72 ^{o}C for 5 min. Barcoded rRNA amplicons were visualized and quantified by agarose gel electrophoresis.

Library Preparation and Sequencing by MinION

MinION library construction employed the 1D sequencing kit (SQK-LSK108- Oxford Nanopore; Oxford England). Two 12 barcoded amplicons (1800 ng total in each) were combined, end-repaired, dA-tailed as per ONT instructions using NEB kits (New England Biolabs, Ipswich, MA, USA) and the modified Ampure bead purification described above. Ligation of the ONT adaptor employed the Blunt/TA ligase master mix (NEB) with an addition of 1 µL of freshly prepared ATP solution (~4 mg/mL) to facilitate ligation. All libraries were analyzed on R9.4 flow cells. To determine the contribution of rRNA operon sequences from PCR reagents and ONT sequencing kits, it was necessary to sequence the PCR negatives from our amplifications. Unfortunately, the original PCR negatives were accidently discarded and the LSK 108 kit that we used for these particular studies is no longer commercially available. Therefore, in order to address this "kitome" issue, we returned to the original DNA from Subject 15 (BAL, throat, mouth, and nose), re-amplified as described in the methods section, and performed a sequencing reaction on both PCR negative and PCR positive samples with the LSK 109 kit. Analysis of this "kitome" indicated a 4000-fold difference in sequence read numbers passing QA/QC (13-Neg reads vs. 41,135-Pos reads) representing a possible contamination of 0.03% (Suppl. Fig 5).

Quality control

BAL, throat swab, mouth wash, and nasal swab samples were collected from 6 subjects, DNA was extracted, and rRNA operons were amplified (with universal rRNA operon primers and barcode primers). Unfortunately, 1 lavage sample from Subject 1 failed to properly amplify (Suppl. Fig 1) and the remaining respiratory samples from this subject were included in overall community analysis but the samples from this particular

subject were not characterized for lung enrichment by relative abundance. A total of ~1.8 x10^6 raw reads were obtained, of which ~1.2 x 10^6 reads passed Albacore basecalling and were separated by barcode. After size selection (3.7-5.7 kb), a total of 623,271 barcoded sequences were screened against the EZ Biocloud database by Discontinuous MegaBlast (Suppl. Fig 2). Of these BLASTED reads, a total of 599,053 sequences passed an additional QA/QC step, having an identity between 70-95% and an alignment with >1200 bp of the 16S rRNA genes in the database (Suppl. Fig. 3).

Data validation

The BLAST screening indicated the respiratory tract was dominated by 5 phyla: *Firmucutes, Proteobacteria, Actinobacteria, Bacteroidetes,* and *Fusobacteria* (representing over 98% of the QA/QC reads) (Fig. 2A). The number of different species within the top 5 genera of these abundant phyla are presented in Fig 2B while the relative abundances of the 15 most abundant genera within the dominant phyla are presented in Fig 3. The relative abundance data indicate the Firmicutes are mostly *Streptococcus* and *Veillonella* genera in lavage for the various subjects. The Protobacteria are largely *Campylobacter* and *Neisseria* genera, with the exception of the lavage samples from subject 7 (*Pseudomonas*) and subject 8 (*Pantoea*). The Actinobacteria are mainly *Actinomyces* in subjects 6, 12, and 15 and *Propionibacteria* or other bacteria in lavage from subjects 7 and 8. While the Bacteroidetes were dominated by *Prevotella* genera. Overall, the rRNA operon profiling detected ~3600 bacterial species with over 750 species present within the dominant phyla. The most abundant bacterial species across all respiratory tract samples were *Veillonella dispar, Streptococcus parasanguinis, Streptococcus infantis, Streptococcus mitis* and

Veillonella atypica. Interestingly, the lavage profiles from Subjects 7 and 8 were markedly different than Subjects 6, 12, and 15 for the Proteobacteria and the Actinobacteria, suggesting these subjects were experiencing a lung infection at the time of sampling.

To assess if the overall lung microbiome differed from throat, nose or mouth microbiome, the data were initially subjected to principle component analysis based on Bray-Curtis dissimilarity index. PCA analysis included log counts data for family and species level resolutions. There was no clear separation between lung and throat, nose, or mouth (PC1-18%, PC2-12% for species level resolution) (Suppl. Fig 4), indicating any differences between the microbial communities is minor. The throat microbiome, compared to nose and mouth, was found to be the most similar to lung microbiome with Bray-Curtis dissimilarity index averaging 0.68 ± 0.21 (species level resolution) and 0.66 ± 0.23 (family level resolution) across all subjects. We also examined if the samples from the different respiratory tract compartments differed for the individual subjects. Similarly, there was no clear separation of bacterial community at the different compartments between the subjects (PC1-26%, PC2-14% for species level resolution; data not shown).

To identify lung-enriched bacteria genera and species, we subtracted the read counts of mouth and nose from bronchial lavage counts after normalization for each subject. Over 1300 lung-enriched bacterial species were discerned across all samples. However, most of these differences in read counts were <50 which may represent methodological variation in raw read results from MinION sequencing. Our prior work has shown that replicate read numbers of >100 have a coefficient of variation of ~12%

or less [39]. Therefore, a conservative threshold of 150 read differences was used to define those bacteria enriched in the lower respiratory tract. This yielded 114 bacterial species from all subjects with a stronger rRNA operon signal in bronchial lavage compared with the higher respiratory tract samples (Suppl. Table 1). To determine whether comparable lung-enrichment was observed for the subjects for particular OTUs, a heat map was generated using the lung-mouth and lung-nose read differences in relative abundances which were >150 reads (Fig. 4). Overall, those lung-enriched OTUs were nearly equally in the bronchial lavage samples for subject 6, 12, and 15. The predominant lung-enriched bacterial genera for this group were *Veillonella spp*., *Prevotella spp*., *Campylobacter spp*., *Actinomyces spp*., and *Megasphaera micronuciformes*. In contrast, subject 7 and 8 were largely missing these particular OTUs and were enriched in *Tatumella spp*., *Pseudomonas spp*., *Pantoea spp*, and *Citrobacter youngae*, consistent with a lung infection at the time of sampling. For many of these genera within bronchial lavage, 3-11 different bacterial species were detected. Interestingly, we did not detect any lung-enriched bacterial species that were present in all subjects. In addition, almost all bacteria species detected in the lung samples are also detected in the throat samples.

To verify we can detect bacterial species which are in higher abundance in the lung, we compared reads across all 4 respiratory compartments. For *Veillonella spp* (the most abundant lung-enriched species in Subj 6, 12, and 15), *V. dispar, V. atypica, V. tobetsuensis, and V. rogosae* generally demonstrated a higher relative abundance in lung samples compared to mouth and nose samples while the throat swab represented an intermediate relative abundance (Fig. 5). For subject 7 and 8, a different pattern was

observed, the *Veillonella* reads for the lung were suppressed or absent. For example, *V. tobetsuensis* was not detected in lung samples from either subject 7 or 8, while *V. rogosae* was absent from subject 7 and in very low abundance for subject 8. Interestingly, the throat/mouth/nose samples for *Veillonella* spp. in these subjects were all higher than the lung samples. Conversely, those bacterial species which yielded a negative number when subtracting upper respiratory samples from bronchial lavage samples (mouth/nose enriched) also displayed an intermediate signal for throat samples for subject 6, 12, and 15 (Fig. 6). For example, the relative abundance for *Streptococcus infantis*, *S. parasanguinis*, and *S. oralis* generally displayed an outside-in pattern for subjects 6, 12, and 15. While subject 7 displayed higher abundances in the lung for *S. infantis*, consistent with a lung infection.

Discussion

DNA-based microbial analysis have identified changes in the human respiratory microbiome for many lung diseases [10-23]. Most of these earlier studies utilized 2 end-member sites (e.g. lung and mouth) to characterize the respiratory microbiome. For example, lung bacterial communities were found in lower abundance compared to the upper respiratory tract [25] and differences between lung and upper respiratory bacterial communities have been described at the genus:family:phylum level [29, 33, 43]. However, because of the low biomass within the lung, end-member analysis to determine the microbial differences along the respiratory tract is difficult to verify. Furthermore, studies resolving only from the bacterial genus to phylum levels will not detect differences within bacterial species or strain levels from the lung. In this study, near full-length rRNA operon sequence reads were utilized to discern those bacteria

capable of colonizing the lung from those being passively transported and eliminated by processes which clear the respiratory tract. Our long-read approach allowed for both species-level detection of bacteria and the assessment of relative abundances along the respiratory tract to distinguish bacterial species enriched in lung samples. Additionally, the inclusion of throat samples represents an intermediate location which enabled verification of relative changes in microbiome communities along the respiratory tract. The results demonstrate that less than 5% of bacterial species detected in the respiratory tract were enriched in the lung.

It is thought that in healthy individuals, the lung microbiome generally becomes inoculated by bacteria from the mouth and the community is maintained by the balance between immigration, colonization, and elimination processes [26]. In contrast, this balance in the “healthy” lung microbiome becomes disturbed during lung infection and diseases [3]. In our study, we can observe a comparable displacement of the lung-enriched microbiome observed in subjects 6, 12, and 15 by the lung-enriched community in subjects 7 and 8. Specifically, high relative abundances of *Pseudomonas spp.* in subject 7 and *Tatumella and Pantoea spp.* in subject 8 was accompanied by a decrease in the relative abundances of *Actinomyces*, *Campylobacter*, *Prevotella*, and *Veillonella* species within their lungs. Our findings are consistent with other studies which have implicated *P. aeruginosa, T. pytseos, and* other Proteobacteria in chronic lung diseases [19], [44, 45], cystic fibrosis [46], or asthma [47]. Likewise, our findings are in agreement with prior work which identified *Veillonella* spp. as one of the most abundant bacteria in the respiratory tract of healthy individuals [48] or with *Prevotella spp*. as prevalent commensal colonizers of mucosal surfaces [49] and members of the

"healthy" lung microbiome [19, 25]. Finally, an important caveat of this study is that our samples were collected at a single time point to distinguish those bacteria displaying a change in relative abundance along the respiratory tract. It would be helpful for future studies to sample the various respiratory compartments over time to delineate changes in the microbiome before, during and after lung infections to monitor lung microbiome dynamics.

Re-use potential

Our study found over 100 different bacterial species which are capable of colonizing the human lung and followed an inside-out distribution with respect to upper respiratory samples. Understanding which specific bacteria can colonize the lower respiratory tract will help in discerning which microbiota constitute a "healthy lung microbiota" and provide a diagnostic tool for studying the role of the microbiome in the development of lung-related diseases.

Availability of supporting data and materials

All raw sequence data is currently being made available at NCBI SRA (Bioproject # PRJNA564314). Further supporting data are available in the *GigaScience* repository, GigaDB [50].

Abbreviations

BAL: bronchoalveolar lavage; OTU: operational taxonomic unit; PCA: principle component analysis; PCR: polymerase chain reaction; ONT: Oxford nanopore

technologies; rRNA: Ribosomal RNA; NCBI: National center for biotechnology information; SRA: sequence read archive

Consent for publication-All authors of the manuscript have read and agreed to its content and are accountable for the accuracy and integrity of the manuscript.

Competing interests-All authors declare there are no competing interests.

Funding-This research was funded in part by an NIEHS Training Grant in Exposure Science 1T32ES019854 to CPW, a Rutgers University Center for Environmental Exposure and Disease (CEED) Pilot Project Grant (#5P30ES005022) to CPW and LJK, and by Rutgers University Indirect Cost Return to LJK.

Authors' contributions -CPW, SH, LRM, and LJK conceived and designed the experiments. SH and associated post-doctoral students collected the respiratory samples. LJK performed DNA extractions. OI amplified the rRNA operons and created the sequence libraries with LJK. LJK performed the sequencing and developed the data analysis approach with LRM and OI. SL and YW performed the principal components analysis. OI, LJK, LRM, and CPW discussed the findings and interpreted the results. OI and LJK wrote the first draft. All authors read, edited, and approved the final manuscript.

Figure Legends:

Figure 1: Location of respiratory samples collected in this study (A) and conceptual model of relative abundance patterns within the respiratory tract (B).

Figure 2: Relative abundance of bacterial phyla within respiratory samples for the various subjects as indicated (A) and the number of bacterial species with the dominant genera/phyla across all subjects (B).

Figure 3: Relative abundance of the top 15 genera within the dominant phyla across all subjects as indicated.

Figure 4: Heat map of lung enriched bacterial species (i.e. lung reads-mouth reads or lung reads-nose reads as indicated) for the various subjects. The number of bacterial species within specific genera are indicated. Full description of lung-enriched taxa is presented in Supplemental Table 1.

Figure 5: Histogram of normalized reads for the respiratory compartments of the different subjects for *Veillonella* spp. as indicated.

Figure 6: Histogram of normalized reads for the respiratory compartments of the different subjects for *Streptococcus* spp. as indicated.

Supplemental Fig. 1: Agarose gel showing amplification of rRNA operons from subjects 1, 6, and 12 as indicated.

Supplemental Fig. 2: Summary data of read numbers for all subjects using the MinION platform.

Supplemental Fig. 3: Plot of percent identity vs. alignment length for MinION raw reads against the EZ BioCloud database using Discontinuous Megablast.

Supplemental Fig. 4: PCA plot of samples located in different compartments from the various subjects based on Bray-Curtis dissimilarity.

Supplemental Fig. 5: Kitome analysis- agarose gel showing amplification of rRNA operons from subject 15 (PCR negatives/positives as indicated) (A) and histogram of reads passing QA/QC for PCR negatives/positives (B).

Supplemental Table 1. Heat map of lung-enriched taxa.

References:

1. Moffatt, M.F. and W.O. Cookson, *The lung microbiome in health and disease.* Clin Med (Lond), 2017. **17**(6): p. 525-529.
2. Huang, Y.J., et al., *The role of the lung microbiome in health and disease. A National Heart, Lung, and Blood Institute workshop report.* Am J Respir Crit Care Med, 2013. **187**(12): p. 1382-7.
3. Mathieu, E., et al., *Paradigms of Lung Microbiota Functions in Health and Disease, Particularly, in Asthma.* Front Physiol, 2018. **9**: p. 1168.
4. Dickson, R.P. and G.B. Huffnagle, *The Lung Microbiome: New Principles for Respiratory Bacteriology in Health and Disease.* PLoS Pathog, 2015. **11**(7): p. e1004923.
5. Chotirmall, S.H., et al., *Microbiomes in respiratory health and disease: An Asia-Pacific perspective.* Respirology, 2017. **22**(2): p. 240-250.
6. O'Dwyer, D.N., R.P. Dickson, and B.B. Moore, *The Lung Microbiome, Immunity, and the Pathogenesis of Chronic Lung Disease.* J Immunol, 2016. **196**(12): p. 4839-47.
7. Shukla, S.D., et al., *Microbiome effects on immunity, health and disease in the lung.* Clin Transl Immunology, 2017. **6**(3): p. e133.
8. Dickson, R.P., et al., *Bacterial Topography of the Healthy Human Lower Respiratory Tract.* MBio, 2017. **8**(1).
9. Qin, S., et al., *Presence of Tropheryma whipplei in Different Body Sites in a Cohort of Healthy Subjects.* Am J Respir Crit Care Med, 2016. **194**(2): p. 243-5.
10. Harris, J.K., et al., *Molecular identification of bacteria in bronchoalveolar lavage fluid from children with cystic fibrosis.* Proc Natl Acad Sci U S A, 2007. **104**(51): p. 20529-33.
11. Rogers, G.B., et al., *characterization of bacterial community diversity in cystic fibrosis lung infections by use of 16s ribosomal DNA terminal restriction fragment length polymorphism profiling.* J Clin Microbiol, 2004. **42**(11): p. 5176-83.
12. Armougom, F., et al., *Microbial diversity in the sputum of a cystic fibrosis patient studied with 16S rDNA pyrosequencing.* Eur J Clin Microbiol Infect Dis, 2009. **28**(9): p. 1151-4.
13. Muhlebach, M.S., et al., *Initial acquisition and succession of the cystic fibrosis lung microbiome is associated with disease progression in infants and preschool children.* PLoS Pathog, 2018. **14**(1): p. e1006798.
14. Carmody, L.A., et al., *The daily dynamics of cystic fibrosis airway microbiota during clinical stability and at exacerbation.* Microbiome, 2015. **3**: p. 12.
15. Price, K.E., et al., *Unique microbial communities persist in individual cystic fibrosis patients throughout a clinical exacerbation.* Microbiome, 2013. **1**(1): p. 27.
16. Huang, Y.J., et al., *A persistent and diverse airway microbiota present during chronic obstructive pulmonary disease exacerbations.* OMICS, 2010. **14**(1): p. 9-59.

17. Pragman, A.A., et al., *The lung microbiome in moderate and severe chronic obstructive pulmonary disease.* PLoS One, 2012. **7**(10): p. e47305.
18. Cabrera-Rubio, R., et al., *Microbiome diversity in the bronchial tracts of patients with chronic obstructive pulmonary disease.* J Clin Microbiol, 2012. **50**(11): p. 3562-8.
19. Hilty, M., et al., *Disordered microbial communities in asthmatic airways.* PLoS One, 2010. **5**(1): p. e8578.
20. Huang, Y.J., et al., *Airway microbiota and bronchial hyperresponsiveness in patients with suboptimally controlled asthma.* J Allergy Clin Immunol, 2011. **127**(2): p. 372-381 e1-3.
21. Goleva, E., et al., *The effects of airway microbiome on corticosteroid responsiveness in asthma.* Am J Respir Crit Care Med, 2013. **188**(10): p. 1193-201.
22. Marri, P.R., et al., *Asthma-associated differences in microbial composition of induced sputum.* J Allergy Clin Immunol, 2013. **131**(2): p. 346-52 e1-3.
23. Huang, Y.J., *The respiratory microbiome and innate immunity in asthma.* Curr Opin Pulm Med, 2015. **21**(1): p. 27-32.
24. Man, W.H., et al., *Bacterial and viral respiratory tract microbiota and host characteristics in children with lower respiratory tract infections: a matched case-control study.* Lancet Respir Med, 2019.
25. Charlson, E.S., et al., *Topographical continuity of bacterial populations in the healthy human respiratory tract.* Am J Respir Crit Care Med, 2011. **184**(8): p. 957-63.
26. Morris, A., et al., *Comparison of the respiratory microbiome in healthy nonsmokers and smokers.* Am J Respir Crit Care Med, 2013. **187**(10): p. 1067-75.
27. Segal, L.N., et al., *Enrichment of lung microbiome with supraglottic taxa is associated with increased pulmonary inflammation.* Microbiome, 2013. **1**(1): p. 19.
28. Twigg, H.L., 3rd, et al., *Use of bronchoalveolar lavage to assess the respiratory microbiome: signal in the noise.* Lancet Respir Med, 2013. **1**(5): p. 354-6.
29. Dickson, R.P., et al., *Spatial Variation in the Healthy Human Lung Microbiome and the Adapted Island Model of Lung Biogeography.* Ann Am Thorac Soc, 2015. **12**(6): p. 821-30.
30. Dickson, R.P., J.R. Erb-Downward, and G.B. Huffnagle, *Towards an ecology of the lung: new conceptual models of pulmonary microbiology and pneumonia pathogenesis.* Lancet Respir Med, 2014. **2**(3): p. 238-46.
31. Dickson, R.P., J.R. Erb-Downward, and G.B. Huffnagle, *Homeostasis and its disruption in the lung microbiome.* Am J Physiol Lung Cell Mol Physiol, 2015. **309**(10): p. L1047-55.
32. Venkataraman, A., et al., *Application of a neutral community model to assess structuring of the human lung microbiome.* MBio, 2015. **6**(1).
33. Bassis, C.M., et al., *Analysis of the upper respiratory tract microbiotas as the source of the lung and gastric microbiotas in healthy individuals.* MBio, 2015. **6**(2): p. e00037.
34. Jain, M., et al., *Improved data analysis for the MinION nanopore sequencer.* Nat Methods, 2015. **12**(4): p. 351-6.
35. Jain, M., et al., *Nanopore sequencing and assembly of a human genome with ultra-long reads.* Nat Biotechnol, 2018. **36**(4): p. 338-345.
36. Cusco, A., et al., *Microbiota profiling with long amplicons using Nanopore sequencing: full-length 16S rRNA gene and whole rrn operon.* F1000Res, 2018. **7**: p. 1755.
37. Benitez-Paez, A. and Y. Sanz, *Multi-locus and long amplicon sequencing approach to study microbial diversity at species level using the MinION portable nanopore sequencer.* Gigascience, 2017. **6**(7): p. 1-12.
38. Kerkhof, L.J., et al., *Profiling bacterial communities by MinION sequencing of ribosomal operons.* Microbiome, 2017. **5**(1): p. 116.

39. Yoon, S.H., et al., *Introducing EzBioCloud: a taxonomically united database of 16S rRNA gene sequences and whole-genome assemblies.* Int J Syst Evol Microbiol, 2017. **67**(5): p. 1613-1617.
40. McGuinness, L.M., et al., *Replicability of bacterial communities in denitrifying bioreactors as measured by PCR/T-RFLP analysis.* Environ Sci Technol, 2006. **40**(2): p. 509-15.
41. Lane, D.J., *16S/23S rRNA sequencing. In: Stackebrandt E and Goodfellow M, editors. Nucleic acid techniques in bacterial systematics.* Chichester, England: John Wiley & Sons Ltd, 1991: p. 115-175.
42. Hunt, D.E., et al., *Evaluation of 23S rRNA PCR primers for use in phylogenetic studies of bacterial diversity.* Appl Environ Microbiol, 2006. **72**(3): p. 2221-5.
43. Charlson, E.S., et al., *Assessing bacterial populations in the lung by replicate analysis of samples from the upper and lower respiratory tracts.* PLoS One, 2012. **7**(9): p. e42786.
44. Faure, E., K. Kwong, and D. Nguyen, *Pseudomonas aeruginosa in Chronic Lung Infections: How to Adapt Within the Host?* Front Immunol, 2018. **9**: p. 2416.
45. Mardaneh, J. and M.M. Dallal, *Isolation, identification and antimicrobial susceptibility of Pantoea (Enterobacter) agglomerans isolated from consumed powdered infant formula milk (PIF) in NICU ward: First report from Iran.* Iran J Microbiol, 2013. **5**(3): p. 263-7.
46. Pustelny, C., et al., *Contribution of Veillonella parvula to Pseudomonas aeruginosa-mediated pathogenicity in a murine tumor model system.* Infect Immun, 2015. **83**(1): p. 417-29.
47. Bisgaard, H., et al., *Association of bacteria and viruses with wheezy episodes in young children: prospective birth cohort study.* BMJ, 2010. **341**: p. c4978.
48. de Steenhuijsen Piters, W.A., E.A. Sanders, and D. Bogaert, *The role of the local microbial ecosystem in respiratory health and disease.* Philos Trans R Soc Lond B Biol Sci, 2015. **370**(1675).
49. Larsen, J.M., *The immune response to Prevotella bacteria in chronic inflammatory disease.* Immunology, 2017. **151**(4): p. 363-374.
50. Ibironke O; McGuinness LR; Lu S-E; Wang Y; Hussain S; Weisel CP; Kerkhof LJ: Supporting data for "Species-Level Evaluation of the Human Respiratory Microbiome" GigaScience Database. 2020. http://dx.doi.org/10.5524/100727.

Figure 1
Click here to access/download;Figure;Figure 1.pdf

Fig. 1

Sample locations within the respiratory system

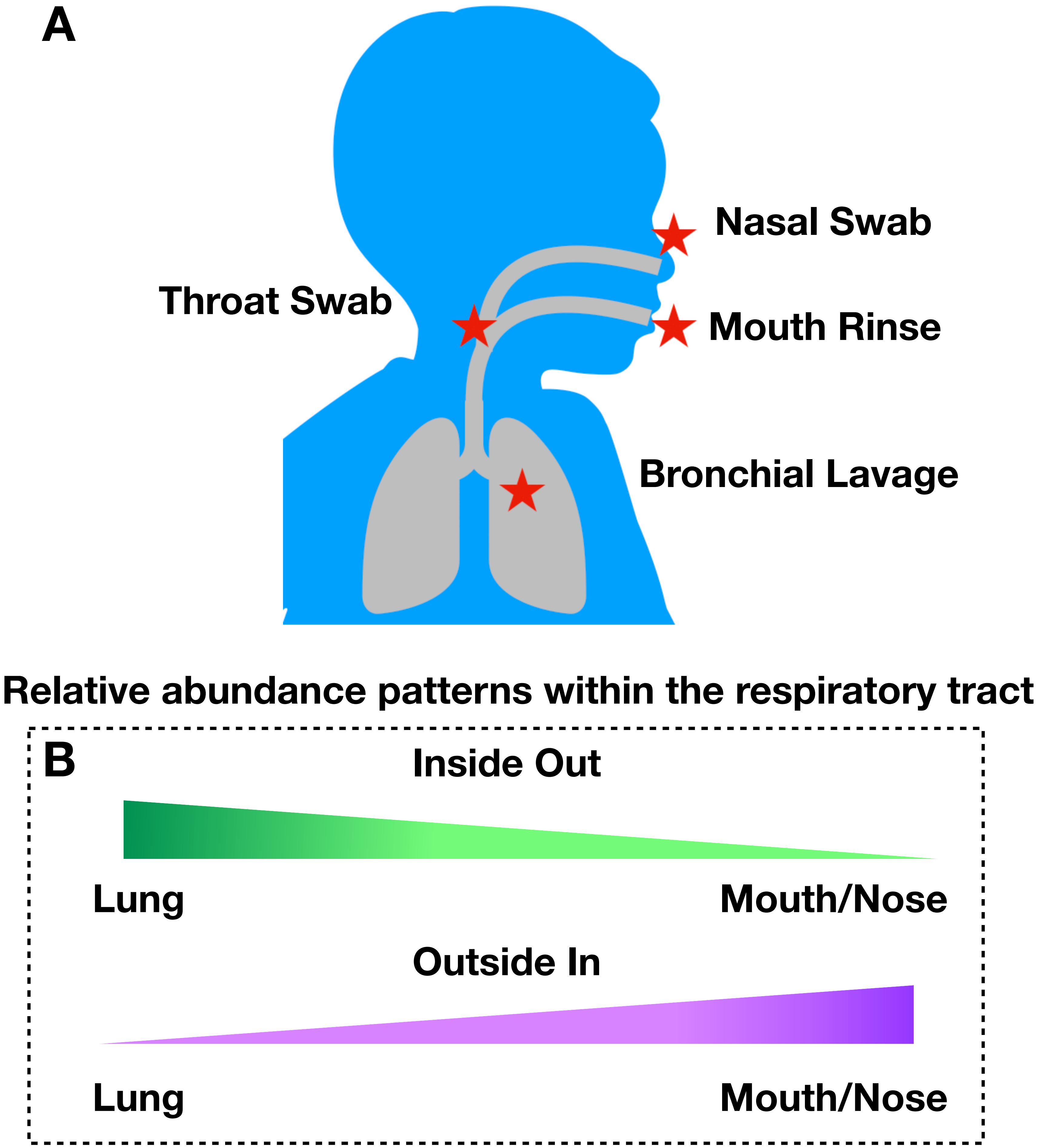

Figure 2

Click here to access/download;Figure;Figure 2.pdf

Fig. 2

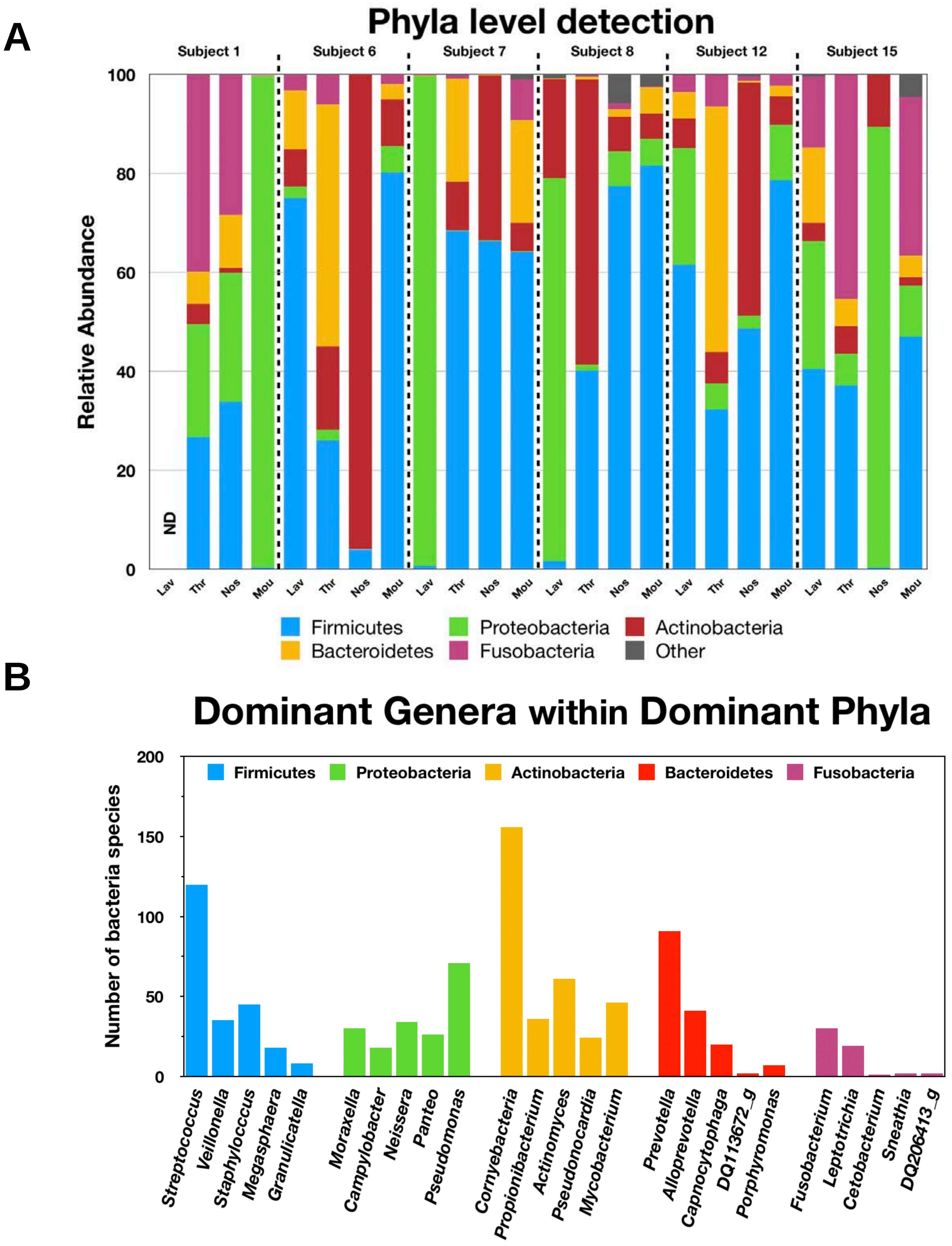

Fig. 3 Relative abundances of the 15 most abundant genera within the dominant phyla

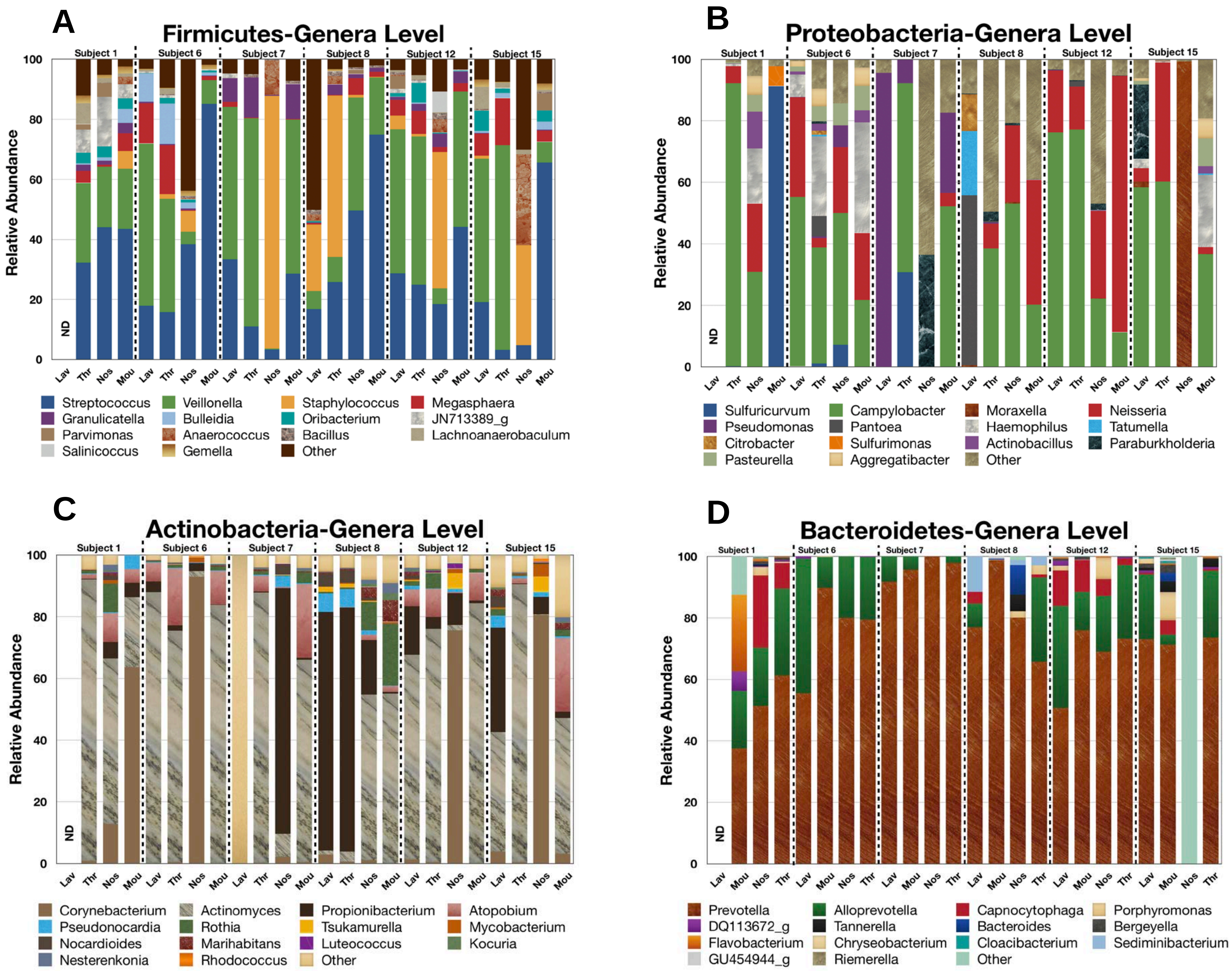

Click here to access/download;Figure;Figure 4.pdf

Fig. 4

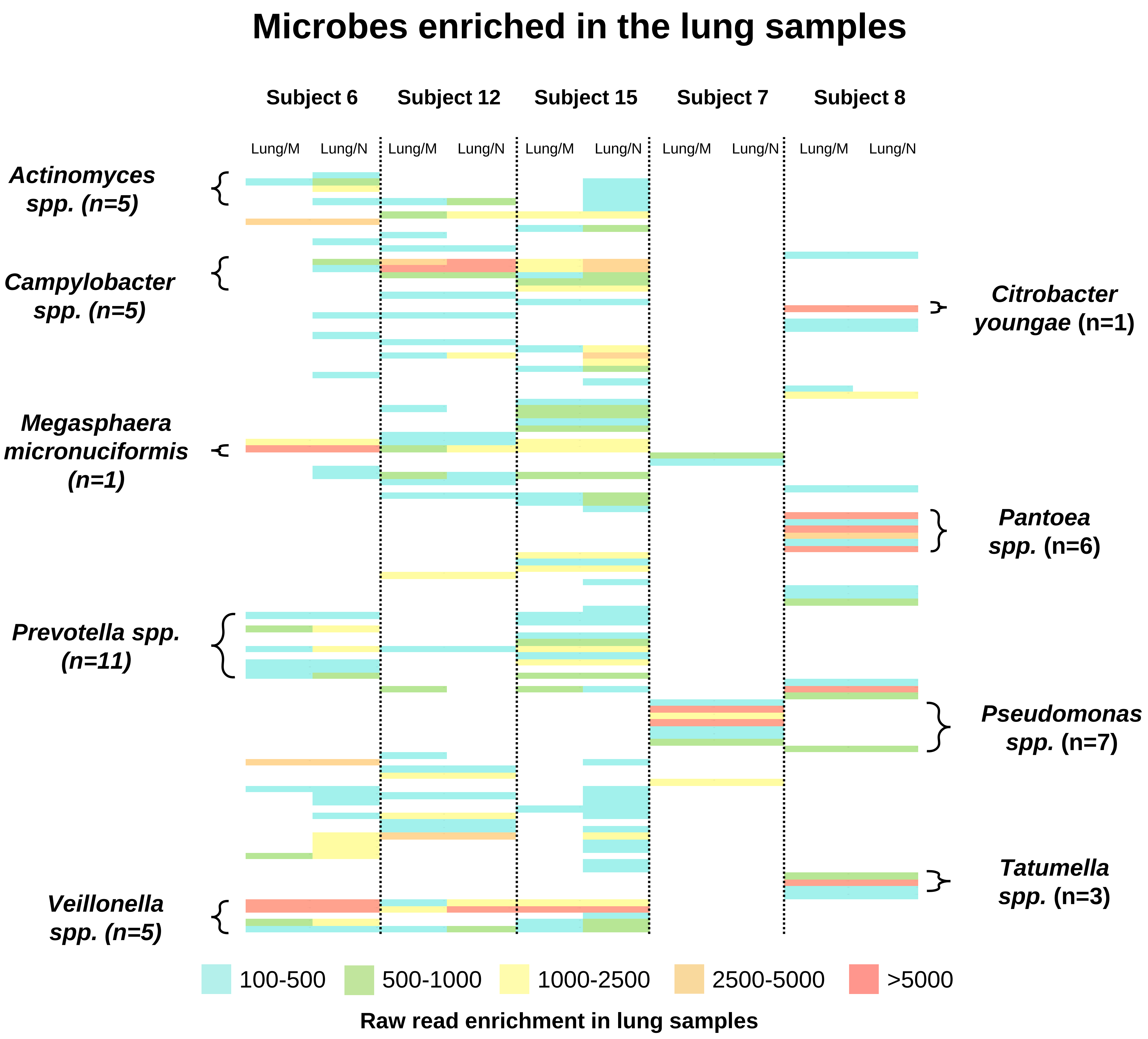

Click here to access/download;Figure;Figure 5.pdf

Fig. 5

Histogram of normalized reads for *Veillonella spp*

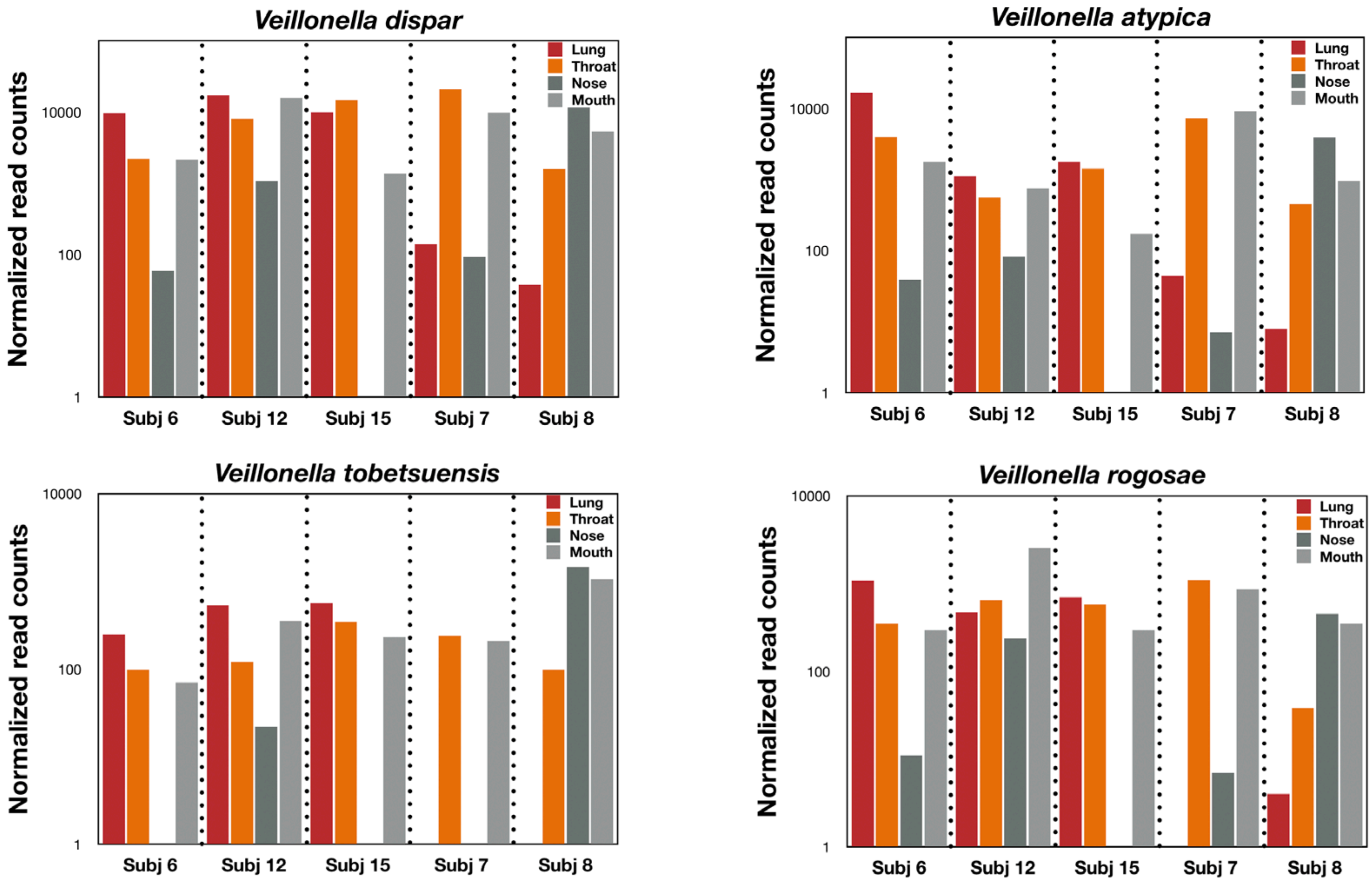

Figure 6
Click here to access/download;Figure;Figure 6.pdf

Fig. 6

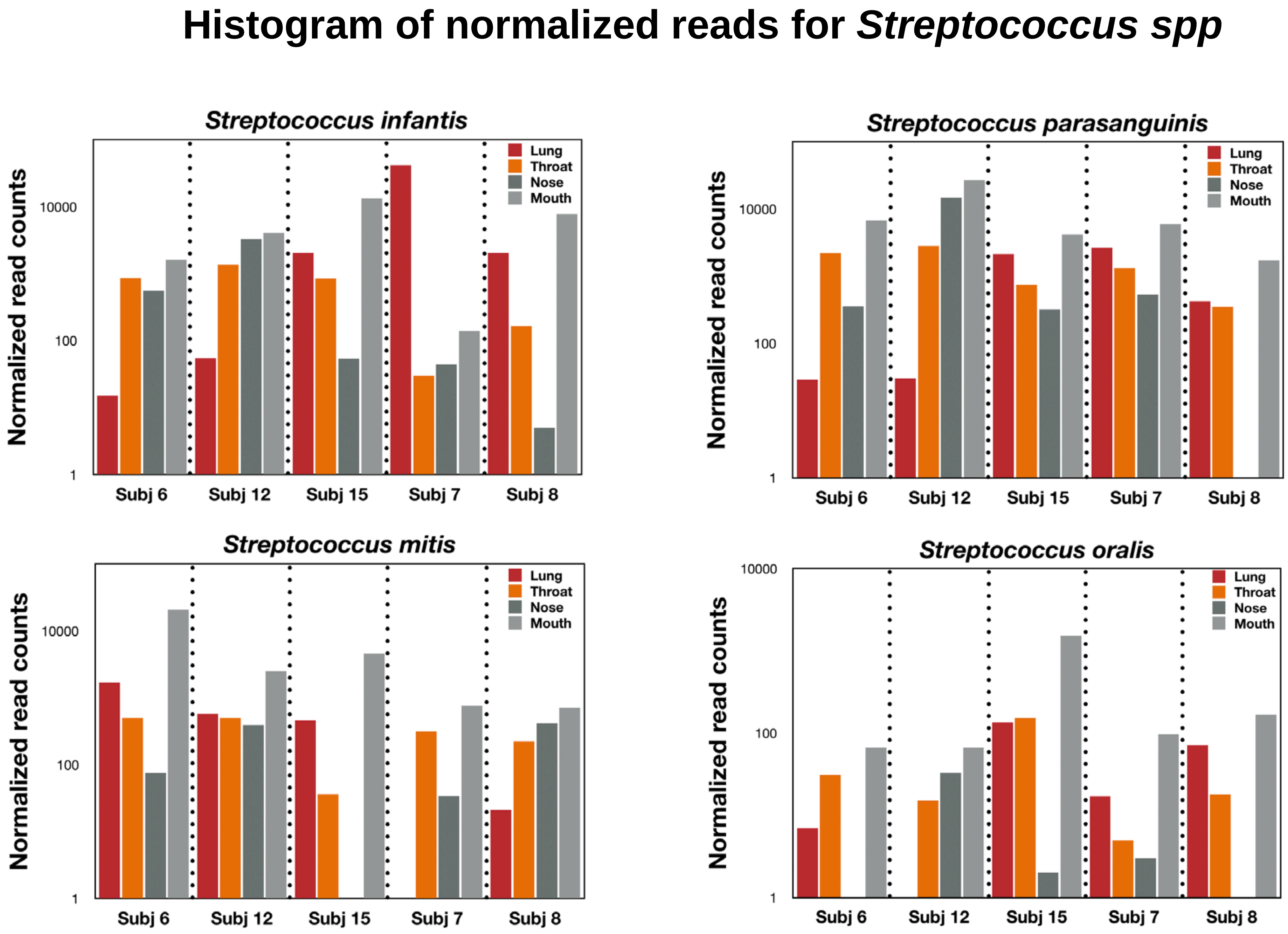

Supplementary Fig. 1

Click here to access/download
Supplementary Material
Suppl Figure 1.pdf

Supplementary Fig. 2

Click here to access/download
Supplementary Material
Suppl Figure 2.pdf

Supplementary Table 1

Click here to access/download
Supplementary Material
Suppl. Table 1 .pdf

Supplementary Fig. 3

Click here to access/download
Supplementary Material
Suppl Fig. 3.pdf

Supplementary Fig. 4

Click here to access/download
Supplementary Material
Suppl Fig. 4.pdf

Supplementary Fig. 5

Click here to access/download
Supplementary Material
Suppl Fig. 5.pdf

Rutgers School of Environmental and Biological Sciences

Lee Kerkhof, Professor
Department of Marine & Coastal Sciences
Rutgers University
71 Dudley Rd.
New Brunswick, NJ 08901-8521

lkerkhof@rutgers.edu
(848) 932-3419
(732) 932-8578 Fax

Rutgers Marine & Coastal Sciences

March 6, 2020

To: Dr. Nicole Nogoy
Re: Manuscript Re-Submission GIGA-D-19-00352

Please consider our revised manuscript, "**Species-Level Evaluation of the Human Respiratory Microbiome"**, for publication in *GigaScience*.

We appreciate the opportunity to address the reviewer concerns. Our responses are detailed below:

Reviewer #1:

Could the authors discuss whether the differences in sampling devices used may explain some of the differences observed?

> Samples were collected by either rinse or swab. We do not observe higher similiarity between throat and nose or lavage and mouth (i.e. collection method). Rather the clustering has more to do with location within the respiratory tract as we had hypothesized and shown in Supplemental Figure 4.

Could they also discuss whether there are limitations regarding the quality of Nanopore sequence are sufficient for specific-level identification?

> In our prior publication, MinION rRNA operon profiling was shown to accurately discern OTUs at the species-level for reads >79% identity, does not generate detectable chimeras, and provides a quantitative response for the top 100, numerically-abundant OTUs (Kerkhof et al., 2017; see below).
>
> We have changed lines 81-84 to reflect this. For the record, Cusco et al., 2018 and Benitez-Paez et al., 2017 have also demonstrated species-level identification using rRNA operons and the MinION platform and are cited in our submission.

L131 - L132 Could the authors provide references for these primers.

> Fixed

L201 - L204 Please provide the range of these values, and it would be good if the authors could compare these values community null model structure.

> There are no range in values for the Principal Components (PC1, PC2) beyond what is presented. We have now included the average and standard deviation for the Bray-Curtis similarity indices across all subjects. We are unsure what null model the reviewer is referring to.

Reviewer #2:
The flowcell type does not appear to be in the manuscript; the specific pore version should be included (e.g. R9.4.1) to make this unambiguous. Ideally it would be in both the Data description and M&M sections

We indicated in the original submission that R9 flow cells were used on line 160. We have updated this text to R9.4 flow cells as requested. R9.4.1 flow cells were not yet available at the time of this study.

The lack of any form of negative control is a serious flaw in the experimental design. At a minimum a no sample control should have been (done) to control for "kitome"

We agree. Unfortunately, the LSK 108 kit that we used for these particular studies are no longer commercially available. However, in order to address the "kitome" issue, we have returned to a subset of the original DNA from Subject 15, amplified as described in the methods section, and performed a sequencing reaction with the LSK 109 kit. Our amplifications are shown here. Both PCR Negatives and PCR Positives were sequenced.

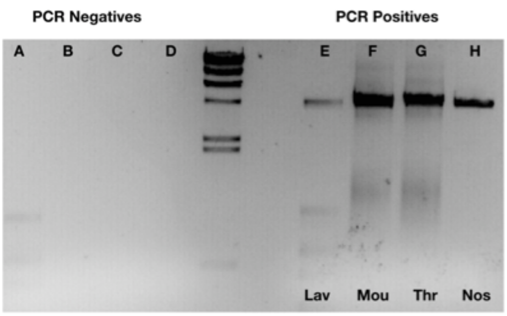

During a 3 hr run, the PCR negatives generated 13 sequences which passed QA/QC while the PCR positives generated over 40,000 sequences which passed QA/QC. This represents a 4000-fold difference in read numbers between negative/positive samples and a possible contamination of 0.03% by the "kitome".

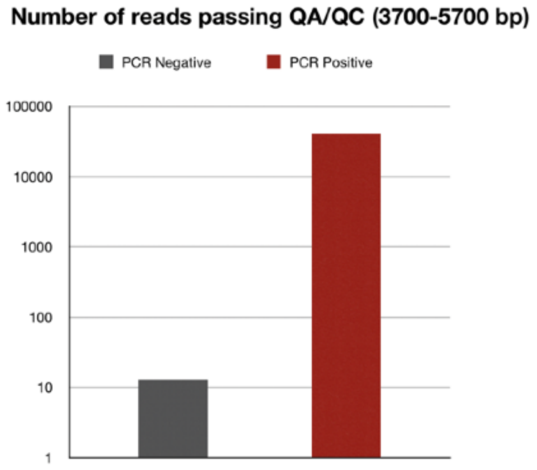

The informatics methodology used to separate the nanopore reads by barcode is not described. It would also be useful to describe how many reads were not assigned a barcode.

Albacore software basecalls and separates MinION reads by barcode as indicated by line 100 in our original submission.

Supplemental Fig 2. illustrates the number of raw reads, those basecalled/barcoded by Albacore, and those reads which pass QA/QC (3700> x >5700 bp).

My major concern is whether the accuracy of nanopore reads is sufficient to make species-level calls bases on rRNA alignments. For example, Veillonella dispar and Veillonella atypica are 98% identical for rRNA, whereas raw nanopore reads have accuracies ranging below 95%. Hence much of the species-level analysis may be artifactual. This is amply illustrated by Supplemental Figure 3.

We disagree with the reviewer's concern here. As cited above, we have demonstrated accurate OTU calling at the species-level for individual MinION ribosomal operon reads >79% identity screened against a 16S rRNA gene database, no detectable chimeras, and a quantitative capability for the top 100, numerically-abundant OTUs using the MinION in our prior publication (Kerkhof et al., 2017).

In our prior study, we did not determine "how low we could go" with respect to accurate OTU calling at the species level. Supplemental Fig. 3 has the 79% identity marked by a dashed line.

In this figure, is "Percent Similarity" really appropriate or is it "Percent Identity" -- are there any mismatches which are scored differently than other mismatches?

Fixed. All mismatches are scored the same.

There is also the concern that MegaBLAST may not be reliable for identifying a top matching sequence depending on parameter settings, a topic which has been discussed (e.g. Shah et al 2018). Tools such as VSEARCH have been explicitly designed for identifying the best match amongst closely related sequences.

We disagree. For the reviewer, we reproduce our in silico testing of 3 different 16S rRNA genes which have been mutated in silico to emulate MinION sequence data (79%-100% identity) from Kerkhof et al., 2017. The table reports the top hit (Description) and the MegaBLAST results. All Discontinuous MegaBlast searches using targets with introduced errors were accurately retrieved from the database at the species (and the strain) levels as shown below. This is possible because the MinION encompasses the entire 16S rRNA gene rather than a short variable region.

Query	Description	Name	Grade	Query coverage	% Pairwise Identity
Sm100	NR_041577.1 Stenotrophomonas maltophilia strain IAM 12423 16S ribosomal RNA gene, complete sequence	9773	100%	100%	100%
Sm99	NR_041577.1 Stenotrophomonas maltophilia strain IAM 12423 16S ribosomal RNA gene, complete sequence	9773	99.5%	100%	98.9%
Sm98	NR_041577.1 Stenotrophomonas maltophilia strain IAM 12423 16S ribosomal RNA gene, complete sequence	9773	98.9%	100%	97.8%
Sm96	NR_041577.1 Stenotrophomonas maltophilia strain IAM 12423 16S ribosomal RNA gene, complete sequence	9773	97.9%	100%	95.8%
Sm94	NR_041577.1 Stenotrophomonas maltophilia strain IAM 12423 16S ribosomal RNA gene, complete sequence	9773	96.8%	100%	93.6%
Sm93	NR_041577.1 Stenotrophomonas maltophilia strain IAM 12423 16S ribosomal RNA gene, complete sequence	9773	96.4%	100%	92.8%
Sm91	NR_041577.1 Stenotrophomonas maltophilia strain IAM 12423 16S ribosomal RNA gene, complete sequence	9773	95.7%	100%	91.4%
Sm90	NR_041577.1 Stenotrophomonas maltophilia strain IAM 12423 16S ribosomal RNA gene, complete sequence	9773	95%	100%	89.9%
Sm89	NR_041577.1 Stenotrophomonas maltophilia strain IAM 12423 16S ribosomal RNA gene, complete sequence	9773	94.5%	100%	88.9%
Sm88	NR_041577.1 Stenotrophomonas maltophilia strain IAM 12423 16S ribosomal RNA gene, complete sequence	9773	94.1%	100%	88.1%
Sm87	NR_041577.1 Stenotrophomonas maltophilia strain IAM 12423 16S ribosomal RNA gene, complete sequence	9773	93.5%	100%	87%
Sm86	NR_041577.1 Stenotrophomonas maltophilia strain IAM 12423 16S ribosomal RNA gene, complete sequence	9773	92.8%	100%	85.5%
SM84	NR_041577.1 Stenotrophomonas maltophilia strain IAM 12423 16S ribosomal RNA gene, complete sequence	9773	92.1%	100%	84.1%
SM83Indel1	NR_041577.1 Stenotrophomonas maltophilia strain IAM 12423 16S ribosomal RNA gene, complete sequence	9773	91.6%	100%	83.1%
SM81Indel2	NR_041577.1 Stenotrophomonas maltophilia strain IAM 12423 16S ribosomal RNA gene, complete sequence	9773	90.7%	100%	81.4%
SM79Indel3	NR_041577.1 Stenotrophomonas maltophilia strain IAM 12423 16S ribosomal RNA gene, complete sequence	9773	90%	100%	79.9%
Cnitrati100	NR_025376.1 Comamonas nitrativorans strain 23310 16S ribosomal RNA gene, partial sequence	11440	100%	100%	100%
Cnitrarti99	NR_025376.1 Comamonas nitrativorans strain 23310 16S ribosomal RNA gene, partial sequence	11440	99.4%	100%	98.9%
Cnitrati98	NR_025376.1 Comamonas nitrativorans strain 23310 16S ribosomal RNA gene, partial sequence	11440	99%	100%	97.9%
Cnitrati96	NR_025376.1 Comamonas nitrativorans strain 23310 16S ribosomal RNA gene, partial sequence	11440	97.8%	100%	95.7%
Cnitrati95	NR_025376.1 Comamonas nitrativorans strain 23310 16S ribosomal RNA gene, partial sequence	11440	97.3%	100%	94.7%
Cnitrati93	NR_025376.1 Comamonas nitrativorans strain 23310 16S ribosomal RNA gene, partial sequence	11440	96.7%	100%	93.3%
cNitrati92	NR_025376.1 Comamonas nitrativorans strain 23310 16S ribosomal RNA gene, partial sequence	11440	95.8%	100%	91.7%
Cnitrati91	NR_025376.1 Comamonas nitrativorans strain 23310 16S ribosomal RNA gene, partial sequence	11440	95.4%	100%	90.9%
Cnitrati90	NR_025376.1 Comamonas nitrativorans strain 23310 16S ribosomal RNA gene, partial sequence	11440	95%	100%	90.1%
Cnitrati89	NR_025376.1 Comamonas nitrativorans strain 23310 16S ribosomal RNA gene, partial sequence	11440	94.4%	100%	88.9%
Cnitriti87	NR_025376.1 Comamonas nitrativorans strain 23310 16S ribosomal RNA gene, partial sequence	11440	93.5%	100%	87%
Cnitrati85	NR_025376.1 Comamonas nitrativorans strain 23310 16S ribosomal RNA gene, partial sequence	11440	92.7%	99.93%	85.4%
Cnitrati81Indel1	NR_025376.1 Comamonas nitrativorans strain 23310 16S ribosomal RNA gene, partial sequence	11440	91.3%	99.94%	82.7%
Cnitrati79Indel2	NR_025376.1 Comamonas nitrativorans strain 23310 16S ribosomal RNA gene, partial sequence	11440	90%	99.94%	80.1%
Cdenit100	NR_025080.1 Comamonas denitrificans strain 123 16S ribosomal RNA gene, partial sequence	11567	97.4%	94.87%	99.9%
Cdenit99	NR_025080.1 Comamonas denitrificans strain 123 16S ribosomal RNA gene, partial sequence	11567	96.7%	94.87%	98.6%
Cdenit97	NR_025080.1 Comamonas denitrificans strain 123 16S ribosomal RNA gene, partial sequence	11567	96%	94.87%	97.2%
Cdenti96	NR_025080.1 Comamonas denitrificans strain 123 16S ribosomal RNA gene, partial sequence	11567	95.3%	94.87%	95.7%
Cdenit95	NR_025080.1 Comamonas denitrificans strain 123 16S ribosomal RNA gene, partial sequence	11567	94.9%	94.87%	95%
Cdenit94	NR_025080.1 Comamonas denitrificans strain 123 16S ribosomal RNA gene, partial sequence	11567	94.3%	94.74%	93.9%
Cdenit92	NR_025080.1 Comamonas denitrificans strain 123 16S ribosomal RNA gene, partial sequence	11567	93.6%	94.74%	92.4%
Cdenit90	NR_025080.1 Comamonas denitrificans strain 123 16S ribosomal RNA gene, partial sequence	11567	92.4%	94.74%	90.1%
Cdenit89	NR_025080.1 Comamonas denitrificans strain 123 16S ribosomal RNA gene, partial sequence	11567	91.9%	94.74%	89%
Cdenit87	NR_025080.1 Comamonas denitrificans strain 123 16S ribosomal RNA gene, partial sequence	11567	90.9%	94.74%	87.2%
Cdenit86	NR_025080.1 Comamonas denitrificans strain 123 16S ribosomal RNA gene, partial sequence	11567	90.5%	94.74%	86.2%
Cdenit85	NR_025080.1 Comamonas denitrificans strain 123 16S ribosomal RNA gene, partial sequence	11567	89.6%	94.74%	84.4%
Cdenit81Indel1	NR_025080.1 Comamonas denitrificans strain 123 16S ribosomal RNA gene, partial sequence	11567	87.9%	94.92%	80.9%
Cdenti79Indel2	NR_025080.1 Comamonas denitrificans strain 123 16S ribosomal RNA gene, partial sequence	11567	87.1%	94.89%	79.3%

The shading and 3D effects for Supplemental Figure 2 are distracting and add nothing. This figure would be improved by removing them.

> We disagree with the reviewer here. This particular figure has been presented on posters at a number of national meetings and the vast majority of people who view our poster comment on the graphics and like them very much.

Supplemental Figure 4 should use a better visual scheme for differentiating the datapoints; the use of soft colors is difficult to disambiguate for individuals with atypical color perception.

> We agree with the reviewer here and have changed the figure to black/white with different symbols for clarity. The ovals have been removed.

Thank you for your consideration.

Sincerely,

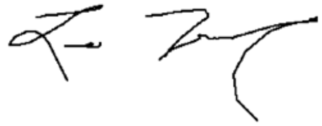

Lee Kerkhof
