# Supplementary material for: Species-level evaluation of the human respiratory microbiome
Source: Gigascience. 2020 Apr 16;9(4):giaa038. doi: 10.1093/gigascience/giaa038 (PMC7162353; doi:10.1093/gigascience/giaa038)
Supplement: giaa038_Supplemental_Files [file giaa038_supplemental_files.zip › Suppl_Table_1.pdf]

	Subj 6		Subj 12		Subj 15		Subj 7		Subj 8	
	Lav-Mou	Lav-Nos	Lav-Mou	Lav-Nos	Lav-Mou	Lav-Nos	Lav-Mou	Lav-Nos	Lav-Mou	Lav-Nos
Actinomyces sp (JRMV)		210								
Actinomyces sp (JVNV)	193	703				196				
Actinomyces sp (KE952139)		1685				174				
Actinomyces_graevenitzii						178				
Actinomyces_odontolyticus		361	256	560		280				
Aggregatibacter_segnis						151				
Alloprevotella sp (FM996479)			984	1103	1497	1553				
Alloprevotella_rava	3204	3402								
Alloprevotella_tannerae					480	521				
Anaerococcus sp (HM587319)			160							
Atopobium_parvulum		238								
Bacillus_altitudinis			311	300						
Buttiauxella_noackiae									191	191
Campylobacter sp (ANNI)		578	4991	5205	2425	3426				
Campylobacter_concisus		265	5596	5723	2476	3649				
Campylobacter_lanienae			922	927	407	579				
Campylobacter_rectus					822	850				
Campylobacter_showae					1583	1642				
Capnocytophaga_sputigena			186	239						
Catonella_morbi					205	423				
Citrobacter_youngae									6378	6378
Clostridium sp (AM420036)		219	393	394						
Enhydrobacter_aerosaccus									186	186
Erwinia_iniecta									157	157
Eubacterium_sulci		226								
Finegoldia_magna			224	224						
Fusobacterium sp (KN173681)					335	1780				
Fusobacterium_nucleatum			245	1097		3667				
Fusobacterium_periodonticum						1451				
Gemella_ sanguinis					357	650				
Granulicatella sp (JVNU)		170								
Haemophilus_parainfluenzae						280				
Ignavigranum sp (GQ072397)									171	
Kluyvera_intermedia									1969	1969
Lachnoanaerobaculum_aburreum					721	806				
Lachnoanaerobaculum_orale			184		562	623				
Lacnoanaerobaculum sp (JH815185)					426	485				
Leptotrichia sp (AF366276)					176	214				
Leptotrichia sp (KI272869)					676	801				
Leptotrichia_trevisanii			172	200						
Leptotrichia_wadei	1616	1680	338	361	1564	1736				
Megasphaera_micronuciformis	6132	6809	667	1566	1749	2007				
Microbulbifer_halophilus							530	530		
Microbulbifer_hydrolyticus							346	346		
Mogibacterium_neglectum		201								
Neisseria_flavescens		443	507	366	574	645				
Neisseria_shayeganii			403	460						
Nocardioides_salarius									473	441
Oribacterium_asaccharolyticum			163	188	409	565				
Oribacterium_parvum					397	854				
Oribacterium_sinus						458				
Pantoea sp (AKIU)									8538	8538
Pantoea_agglomerans									305	305
Pantoea_allii									6933	6933
Pantoea_ananatis									4345	4345

Pantoea_eucrina									212	212
Pantoea_stewartii									8589	8589
Paraburkholderia_fungorum					1188	1188				
Paraburkholderia_ginsengisoli					449	449				
Paraburkholderia_sabiae					2212	2212				
Parvimonas_micra			1776	1776						
Pasteurella_dagmatis						196				
Pectobacterium_wasabiae									182	182
Peptoniphilus_harei									215	166
Pluralibacter_pyrinus									847	847
Prevotella sp (CP003667)						182				
Prevotella sp (FM995711)	346	443			246	258				
Prevotella sp (KI259591)					174	174				
Prevotella_histicola	916	1514								
Prevotella_intermedia					280	320				
Prevotella_jejuni					582	601				
Prevotella_melaninogenica	338	1172	411	497	2263	2488				
Prevotella_nanceiensis					322	378				
Prevotella_pallens	337	475			1932	2101				
Prevotella_salivae	360	518			607	663				
Prevotella_veroralis	218	251								
Propionibacterium sp (AFUN)									216	200
Propionibacterium_acnes			573		711	411			9041	8330
Propionibacterium_granulosum									617	588
Pseudomona_stutzeri							751	751		
Pseudomonas sp (JN208915)							169	169		
Pseudomonas_aeruginosa							54014	54036		
Pseudomonas_benzenivorans							1222	1222		
Pseudomonas_nitroreducens							6829	6840		
Pseudomonas_oryzihabitans							272	272		
Pseudomonas_plecoglossicida							383	383		
Pseudonocardia_ailaonensis									808	752
Selenomonas sp (CP012071)			170							
Solobacterium_moorei	4362	4800				156				
Staphylococcus_argenteus			175	168						
Staphylococcus_simiae			1460	1407						
Stenotrophomonas_maltophilia							1732	1738		
Stomatobaculum_longum	211	494				276				
Streptococcus sp (ASZZ)		356	157	412		214				
Streptococcus sp (JVFV)		228				178				
Streptococcus sp (JYGT)					175	209				
Streptococcus_cristatus		231	1040	1142		454				
Streptococcus_equi			246	265						
Streptococcus_equinus			221	228		329				
Streptococcus_infantis		1990	4031	4126		2042				
Streptococcus_mitis		1624				454				
Streptococcus_parasanguinis		1822				427				
Streptococcus_pseudopneumoniae	850	1347								
Streptococcus_salivarius						174				
Streptococcus_vestibularis						156				
Tatumella_morbirosei									678	678
Tatumella_ptyseos									10182	10181
Tatumella_punctata									186	186
Tsukamurella_inchonensis									222	212
Veillonella_atypica	14940	16683	369	1046	1601	1776				

Veillonella_dispar	7472	9538	1241	15914	8641	9986				
Veillonella_parvula						156				
Veillonella_rogosae	778	1067			403	699				
Veillonella_tobetsuensis	179	249	178	511	339	570				
