# Supplementary figures and images for: Species-level evaluation of the human respiratory microbiome
Source: Gigascience. 2020 Apr 16;9(4):giaa038. doi: 10.1093/gigascience/giaa038 (PMC7162353; doi:10.1093/gigascience/giaa038)

Suppl. Fig. 1

PCR amplifications of rRNA operons from respiratory tract samples

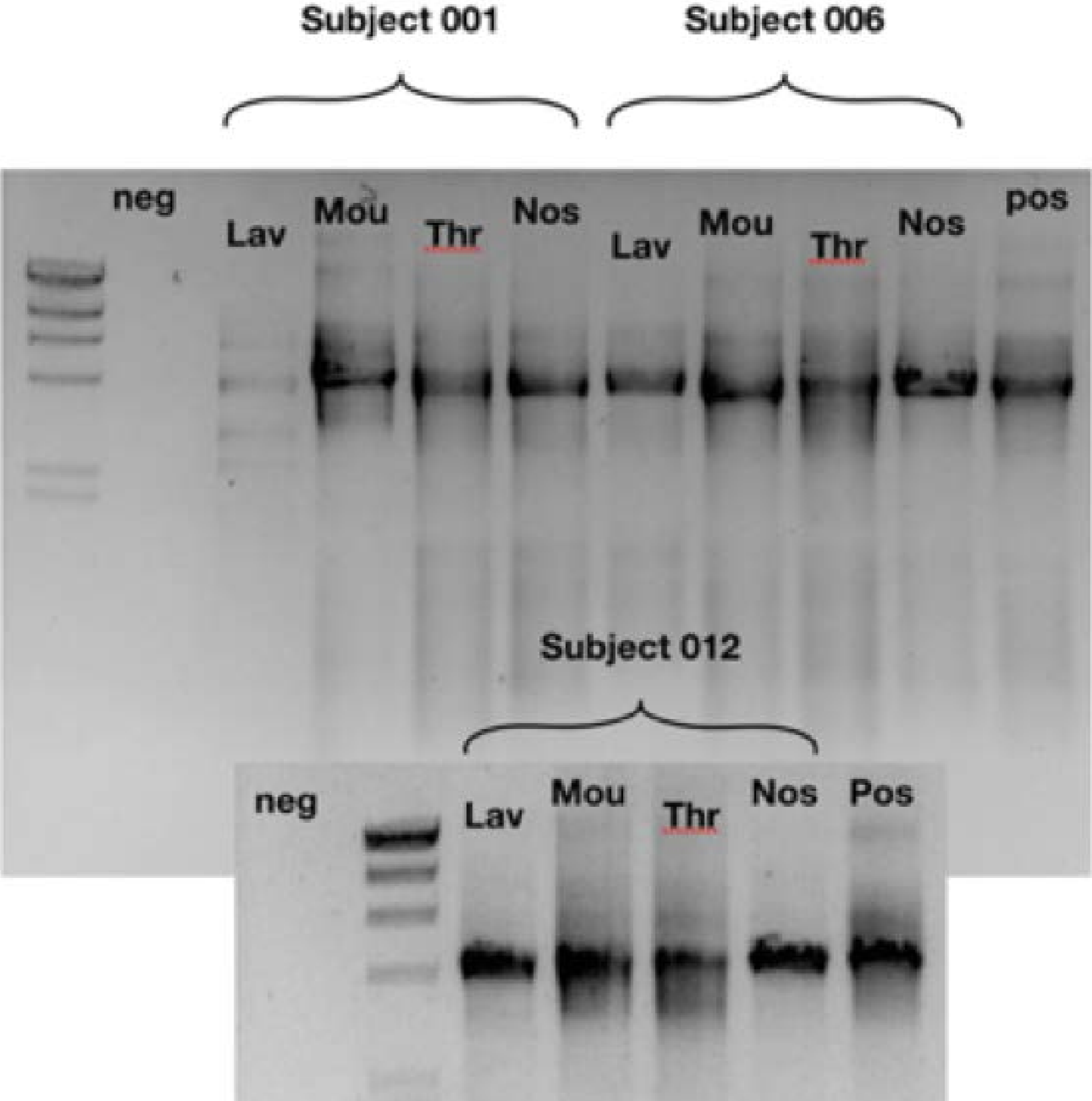

Supplement: giaa038_Supplemental_Files [file giaa038_supplemental_files.zip › Suppl_Figure_1.pdf]

Suppl. Fig. 2

Summary data of read numbers for all subjects using the MinION platform.

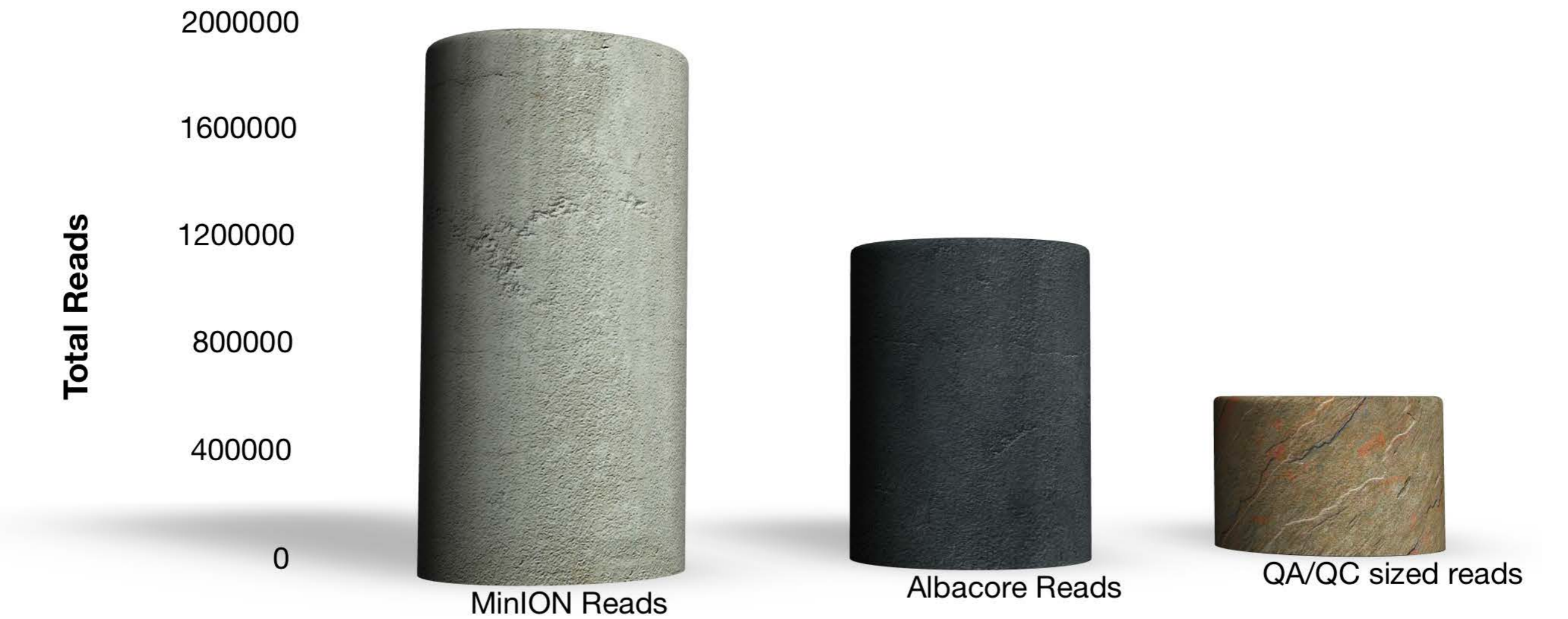

Supplement: giaa038_Supplemental_Files [file giaa038_supplemental_files.zip › Suppl_Figure_2.pdf]

**Suppl. Fig. 3**

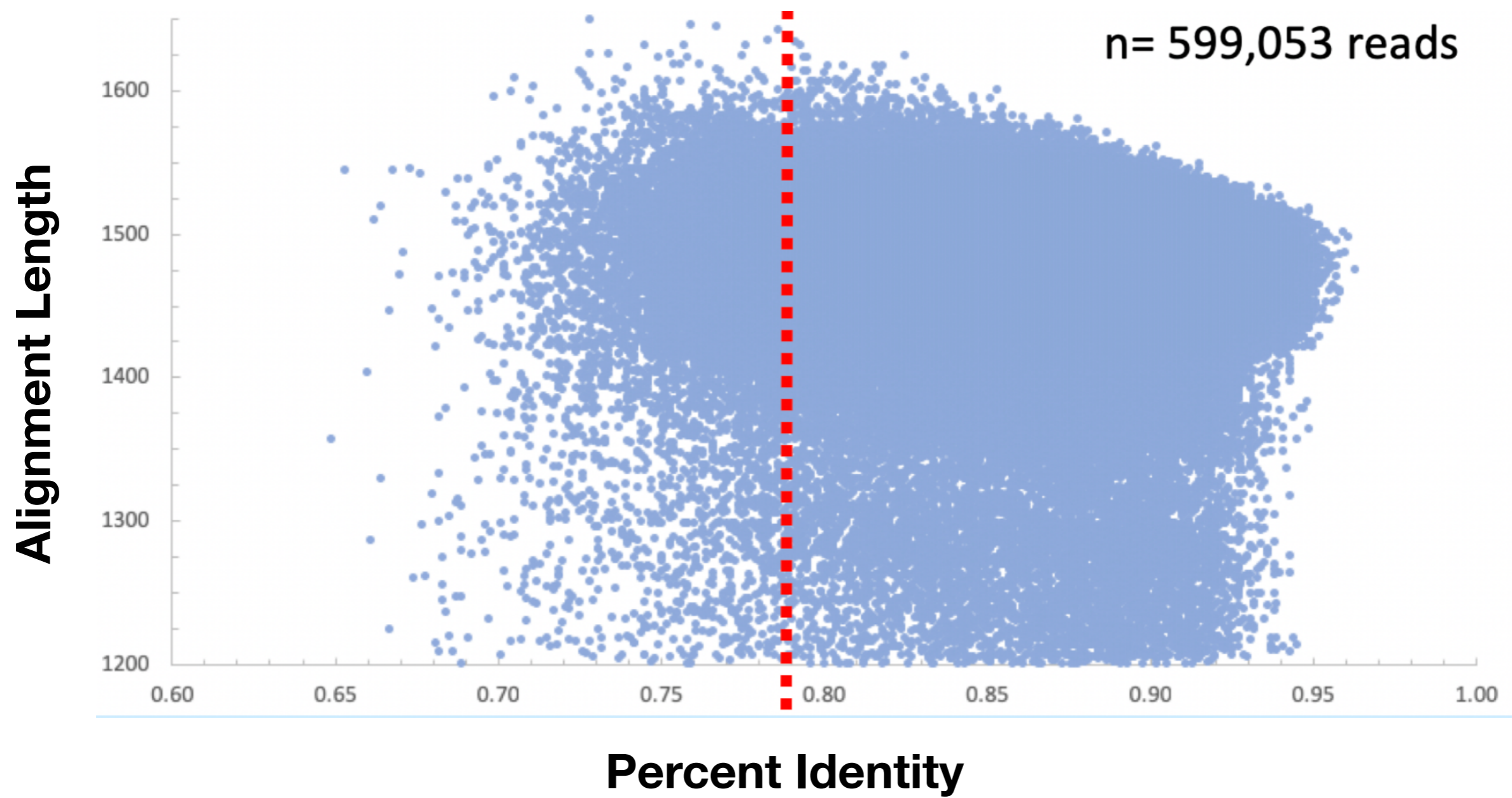

Supplement: giaa038_Supplemental_Files [file giaa038_supplemental_files.zip › Suppl_Figure_3.pdf]

Suppl. Fig. 4

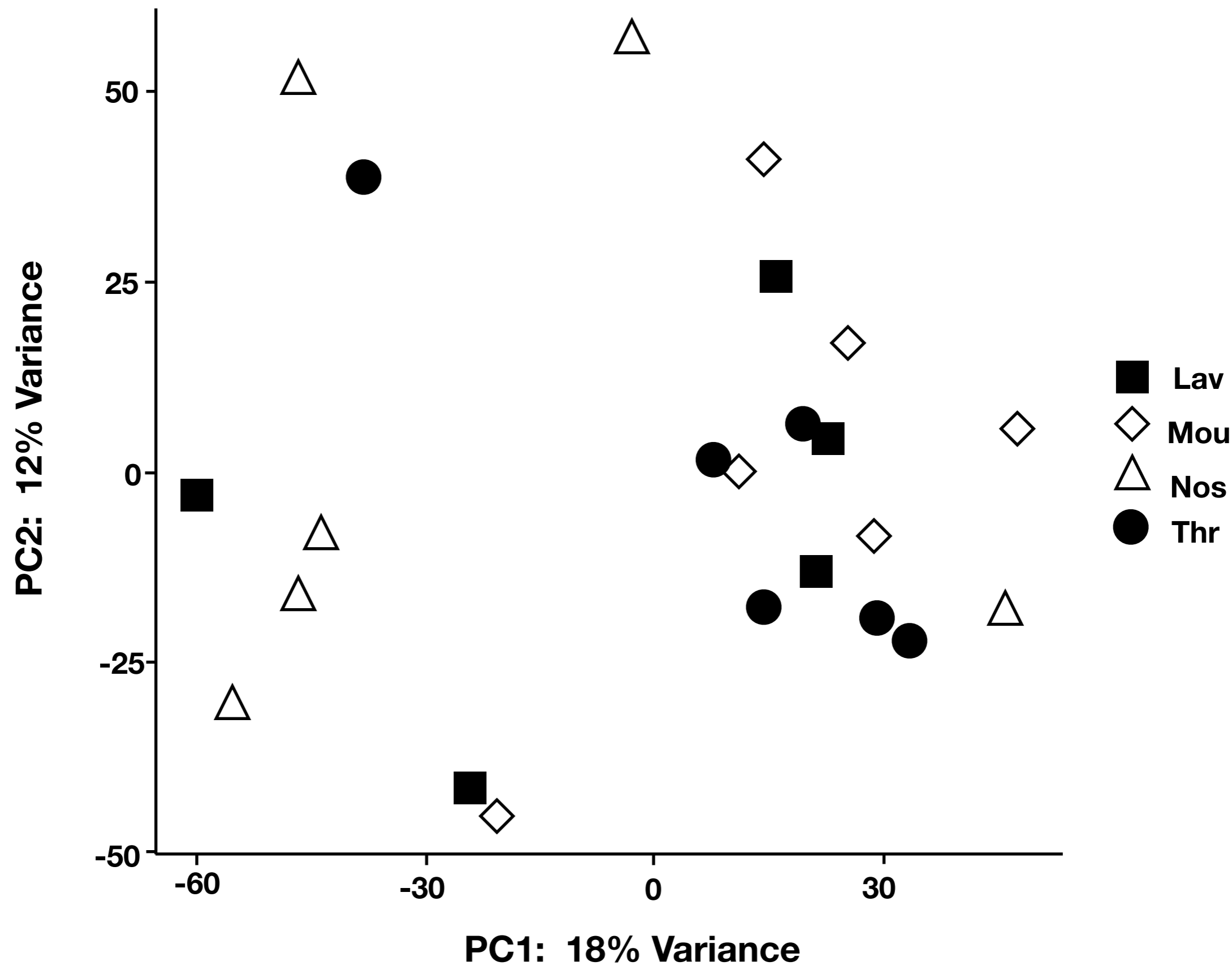

Supplement: giaa038_Supplemental_Files [file giaa038_supplemental_files.zip › Suppl_Figure_4.pdf]

Suppl. Fig. 5

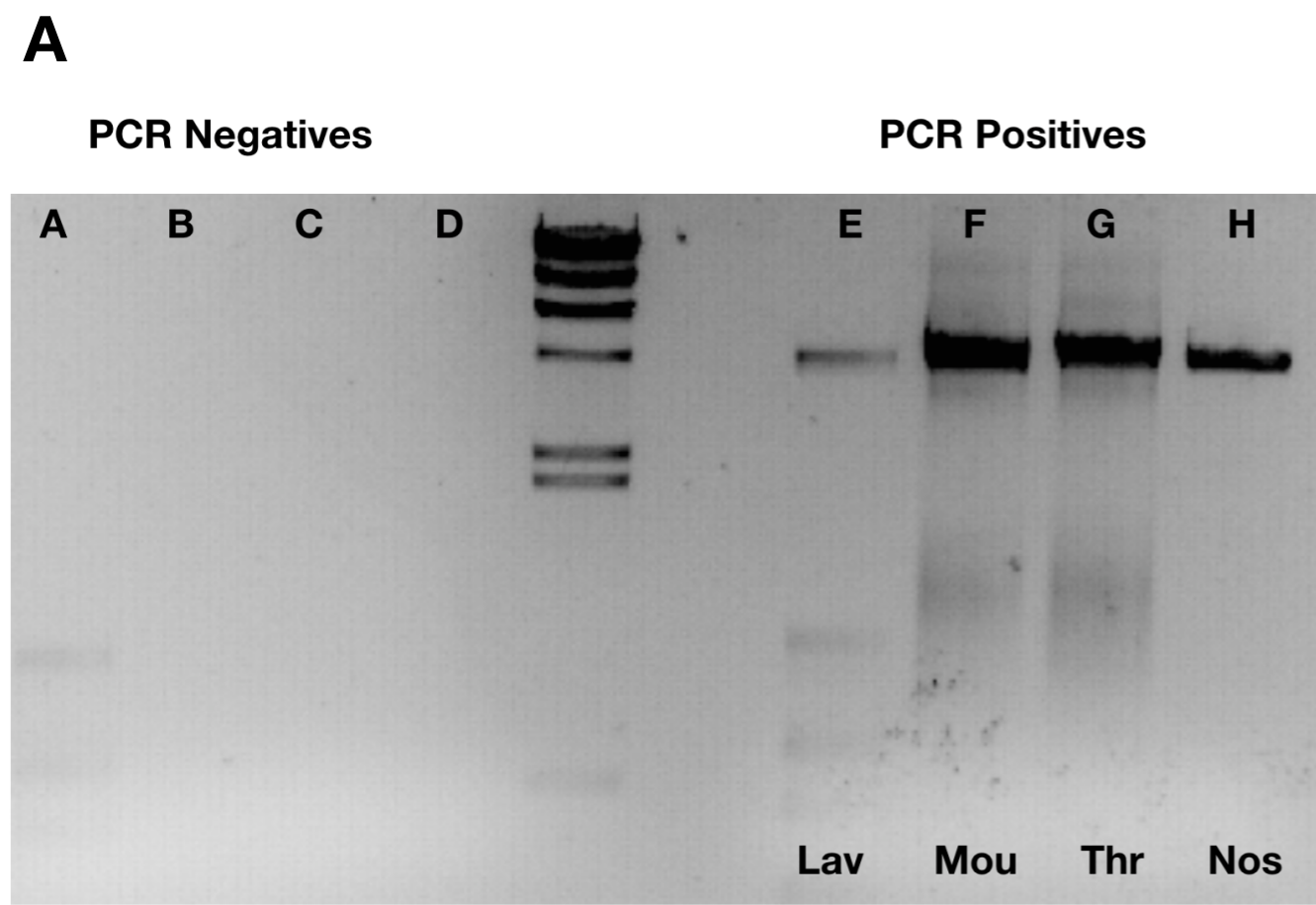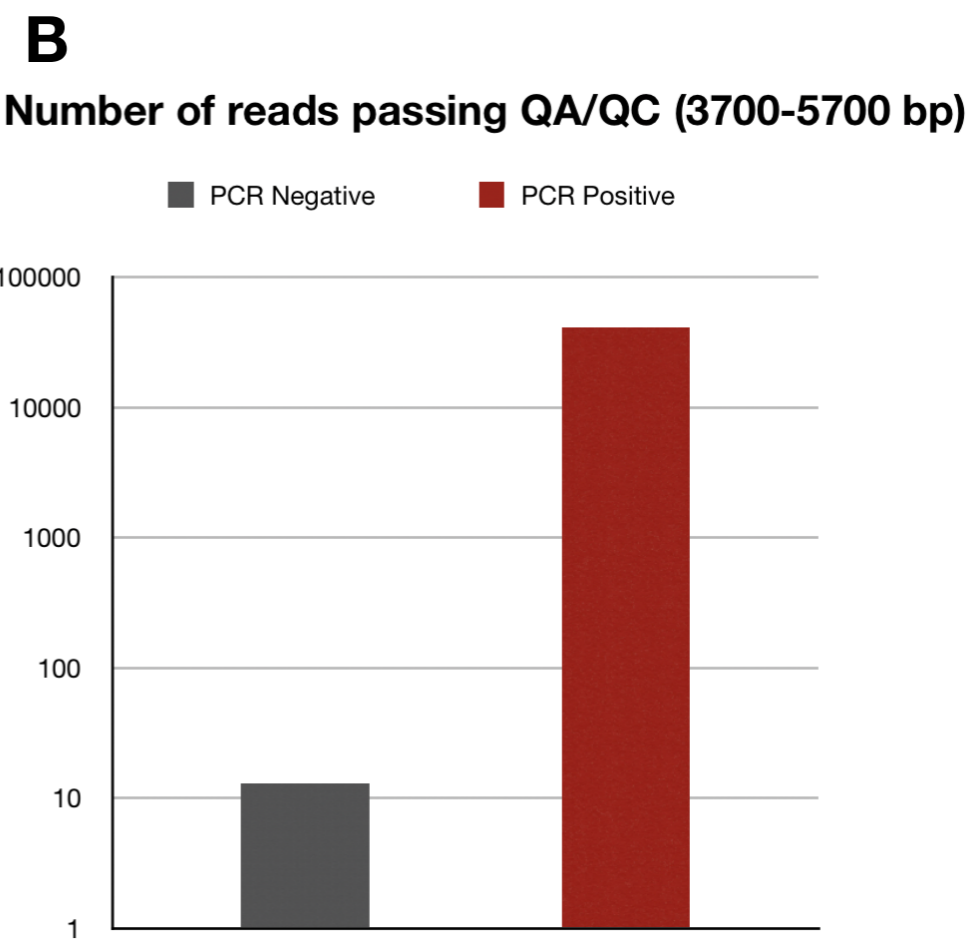

Supplement: giaa038_Supplemental_Files [file giaa038_supplemental_files.zip › Suppl_Figure_5.pdf]
