# Supplementary material for: Species-level evaluation of the human respiratory microbiome
Source: Gigascience. 2020 Apr 16;9(4):giaa038. doi: 10.1093/gigascience/giaa038 (PMC7162353; doi:10.1093/gigascience/giaa038)
Supplement: giaa038_Response-to-Reviewer_Comments_Original_Submission.pdf [file giaa038_response-to-reviewer_comments_original_submission.pdf]

Author's Response To Reviewer Comments

Close

****
Please also see the cover letter which has the response to the reviewers with figures which can not be included in this text box.
****

Response to reviewers

Reviewer #1:

Could the authors discuss whether the differences in sampling devices used may explain some of the differences observed?

Samples were collected by either rinse or swab. We do not observe higher similiarity between throat and nose or lavage and mouth (i.e. collection method). Rather the clustering has more to do with location within the respiratory tract as we had hypothesized and shown in Supplemental Figure 4.

Could they also discuss whether there are limitations regarding the quality of Nanopore sequence are sufficient for specific-level identification?

In our prior publication, MinION rRNA operon profiling was shown to accurately discern OTUs at the species-level for reads >79% identity, does not generate detectable chimeras, and provides a quantitative response for the top 100, numerically-abundant OTUs (Kerkhof et al., 2017).

We have changed lines 81-84 to reflect this. For the record, Cusco et al., 2018 and Benitez-Paez et al., 2017 who have also demonstrated species-level identification using rRNA operons and the MinION platform and our cited in our submission.

L131 - L132 Could the authors provide references for these primers.

Fixed

L201 - L204 Please provide the range of these values, and it would be good if the authors could compare these values community null model structure.

There are no range in values for the Principal Components (PC1, PC2) beyond what is presented. We have now included the average and standard deviation for the Bray-Curtis similarity indices across all subjects. We are unsure what null model the reviewer is referring to. However,

Reviewer #2:
The flowcell type does not appear to be in the manuscript; the specific pore version should be included (e.g. R9.4.1) to make this unambiguous. Ideally it would be in both the Data description and M&M sections

We indicated in the original submission that R9 flow cells were used on line 160. We have updated this text to R9.4 flow cells as requested. R9.4.1 flow cells were not yet available at the time of this study.
The lack of any form of negative control is a serious flaw in the experimental design. At a minimum a no sample control should have been (done) to control for "kitome"

We agree. Unfortunately, the LSK 108 kit that we used for these particular studies are no longer commercially available. However, in order to address the “kitome” issue, we have returned to a subset of the original DNA from Subject 15, amplified as described in the methods section, and performed a sequencing reaction with the LSK 109 kit. Our amplifications are shown here. Both PCR Negatives and PCR Positives were sequenced.

During a 3 hr run, the PCR negatives generated 13 sequences which passed QA/QC while the PCR positives generated over 40,000 sequences which passed QA/QC. This represents a 4000-fold difference in read numbers between negative and positive samples and a possible contamination of 0.03% by the "kitome".

The informatics methodology used to separate the nanopore reads by barcode is not described. It would also be useful to describe how many reads were not assigned a barcode.

Albacore software basecalls and separates MinION reads by barcode as indicated by line 100 in our original submission.

Supplemental Fig 2. illustrates the number of raw reads, those basecalled/barcoded by Albacore, and those reads which pass QA/QC (3700> x >5700 bp).

My major concern is whether the accuracy of nanopore reads is sufficient to make species-level calls bases on rRNA alignments. For example, Veillonella dispar and Veillonella atypica are 98% identical for rRNA, whereas raw nanopore reads have accuracies ranging below 95%. Hence much of the species-level analysis may be artifactual. This is amply illustrated by Supplemental Figure 3.

We disagree with the reviewer's concern here. As cited above, we have demonstrated accurate OTU calling at the species-level for individual MinION ribosomal operon reads >79% identity screened against a 16S rRNA gene database, no detectable chimeras, and a quantitative capability for the top 100, numerically-abundant OTUs using the MinION in our prior publication (Kerkhof et al., 2017).

In our prior study, we did not determine "how low we could go" with respect to accurate OTU calling at the species level. Supplemental Fig. 3 has the 79% identity marked by a dashed line.

In this figure, is "Percent Similarity" really appropriate or is it "Percent Identity" -- are there any mismatches which are scored differently than other mismatches?

Fixed. All mismatches are scored the same.

There is also the concern that MegaBLAST may not be reliable for identifying a top matching sequence depending on parameter settings, a topic which has been discussed (e.g. Shah et al 2018). Tools such as VSEARCH have been explicitly designed for identifying the best match amongst closely related sequences.

We disagree. For the reviewer, we reproduce our in silico testing of 3 different 16S rRNA genes which have been mutated in silico to emulate MinION sequence data (79%-100% identity) from Kerkhof et al., 2017. The table reports the top hit (Description) and the MegaBLAST results. All Discontinuous MegaBlast searches using targets with introduced errors were accurately retrieved from the database at the species (and the strain) levels as shown below. This is possible because the MinION encompasses the entire 16S rRNA gene rather than a short variable region.

The shading and 3D effects for Supplemental Figure 2 are distracting and add nothing. This figure would be improved by removing them.

We disagree with the reviewer here. This particular figure has been presented on posters at a number of national meetings and the vast majority of people who view our poster comment on the graphics and like them very much.

Supplemental Figure 4 should use a better visual scheme for differentiating the datapoints; the use of soft colors is difficult to disambiguate for individuals with atypical color perception. Given that the four sample sites begin with different letters, incorporating these as markers would be ideal, as it would eliminate the need to remap sites to colors or shapes. The significance of the overlaid ovals should be described in the legend.

We agree with the reviewer here and have changed the figure to black/white with different symbols for clarity. The ovals have been removed.

Close
