# Supplementary material for: Species-level evaluation of the human respiratory microbiome
Source: Gigascience. 2020 Apr 16;9(4):giaa038. doi: 10.1093/gigascience/giaa038 (PMC7162353; doi:10.1093/gigascience/giaa038)
Supplement: giaa038_Reviewer_1_Report_Original_Submission — Mohammad Bahram -- 11/19/2019 Reviewed [file giaa038_reviewer_1_report_original_submission.pdf]

Reviewer Report

Title: Species-Level Evaluation of the Human Respiratory Microbiome

Version: Original Submission **Date:** 11/19/2019

Reviewer name: Mohammad Bahram

Reviewer Comments to Author:

This is a rather straightforward manuscript describing an interesting dataset on the human respiratory tract microbiome. The authors used long-amplicon sequencing for characterizing microbial communities in samples taken from various parts of the respiratory tract. They found a number of bacteria to be more abundant and suggest that these could play a functional role on lung health. Although the number of replicate, which were sampled at a single time point, are low for making a sound conclusion, the dataset is unique as it includes samples from different parts of the respiratory tract. I believe the data would be useful for further analyses. I have only a few minor suggestions.
Could the authors discuss whether the differences in sampling devices used may explain some of the differences observed? Could they also discuss whether there are limitations regarding the quality of Nanopore sequence are sufficient for specific-level identification?
L131 - L132 Could the authors provide references for these primers.
L201 - L204 Please provide the range of these values, and it would be good if the authors could compare these values community null model structure.

Level of Interest

Please indicate how interesting you found the manuscript: Choose an item.

Quality of Written English

Please indicate the quality of language in the manuscript: Choose an item.

Declaration of Competing Interests

Please complete a declaration of competing interests, considering the following questions:

- Have you in the past five years received reimbursements, fees, funding, or salary from an organisation that may in any way gain or lose financially from the publication of this manuscript, either now or in the future?
- Do you hold any stocks or shares in an organisation that may in any way gain or lose financially from the publication of this manuscript, either now or in the future?
- Do you hold or are you currently applying for any patents relating to the content of the manuscript?

- Have you received reimbursements, fees, funding, or salary from an organization that holds or has applied for patents relating to the content of the manuscript?
- Do you have any other financial competing interests?
- Do you have any non-financial competing interests in relation to this paper?

If you can answer no to all of the above, write 'I declare that I have no competing interests' below. If your reply is yes to any, please give details below.

I declare that I have no competing interests

I agree to the open peer review policy of the journal. I understand that my name will be included on my report to the authors and, if the manuscript is accepted for publication, my named report including any attachments I upload will be posted on the website along with the authors' responses. I agree for my report to be made available under an Open Access Creative Commons CC-BY license (http://creativecommons.org/licenses/by/4.0/). I understand that any comments which I do not wish to be included in my named report can be included as confidential comments to the editors, which will not be published.

Choose an item.

To further support our reviewers, we have joined with Publons, where you can gain additional credit to further highlight your hard work (see: https://publons.com/journal/530/gigascience). On publication of this paper, your review will be automatically added to Publons, you can then choose whether or not to claim your Publons credit. I understand this statement.

Yes Choose an item.
