# Supplementary material for: Species-level evaluation of the human respiratory microbiome
Source: Gigascience. 2020 Apr 16;9(4):giaa038. doi: 10.1093/gigascience/giaa038 (PMC7162353; doi:10.1093/gigascience/giaa038)
Supplement: giaa038_Reviewer_2_Report_Original_Submission — Keith Robison -- 12/9/2019 Reviewed [file giaa038_reviewer_2_report_original_submission.pdf]

Reviewer Report

Title: Species-Level Evaluation of the Human Respiratory Microbiome

Version: Original Submission **Date:** 12/9/2019

Reviewer name: Keith Robison

Reviewer Comments to Author:

Overall the paper presents a useful set of data generated with the Oxford Nanopore platform on the human respiratory tract.
There are some details of the experiment which would benefit from further exposition.
The flowcell type does not appear to be in the manuscript; the specific pore version should be included (e.g. R9.4.1) to make this unambiguous. Ideally it would be in both the Data description and M&M sections
The lack of any form of negative control is a serious flaw in the experimental design. At a minimum a no sample control should have been to control for "kitome"
The informatics methodology used to separate the nanopore reads by barcode is not described. It would also be useful to describe how many reads were not assigned a barcode.
My major concern is whether the accuracy of nanopore reads is sufficient to make species-level calls bases on rRNA alignments. For example, Veillonella dispar and Veillonella atypica are 98% identical for rRNA, whereas raw nanopore reads have accuracies ranging below 95%. Hence much of the species-level analysis may be artifactual. This is amply illustrated by Supplemental Figure 3. In this figure, is "Percent Similarity" really appropriate or is it "Percent Identity" -- are there any mismatches which are scored differently than other mismatches? There is also the concern that MegaBLAST may not be reliable for identifying a top matching sequence depending on parameter settings, a topic which has been discussed (e.g. Shah et al 2018). Tools such as VSEARCH have been explicitly designed for identifying the best match amongst closely related sequences.
The shading and 3D effects for Supplemental Figure 2 are distracting and add nothing. This figure would be improved by removing them.
Supplemental Figure 4 should use a better visual scheme for differentiating the datapoints; the use of soft colors is difficult to disambiguate for individuals with atypical color perception. Given that the four sample sites begin with different letters, incorporating these as markers would be ideal, as it would eliminate the need to remap sites to colors or shapes. The significance of the overlaid ovals should be described in the legend.

Level of Interest

Please indicate how interesting you found the manuscript: Choose an item.

Quality of Written English

Please indicate the quality of language in the manuscript: Choose an item.

Declaration of Competing Interests

Please complete a declaration of competing interests, considering the following questions:

- Have you in the past five years received reimbursements, fees, funding, or salary from an organisation that may in any way gain or lose financially from the publication of this manuscript, either now or in the future?
- Do you hold any stocks or shares in an organisation that may in any way gain or lose financially from the publication of this manuscript, either now or in the future?
- Do you hold or are you currently applying for any patents relating to the content of the manuscript?
- Have you received reimbursements, fees, funding, or salary from an organization that holds or has applied for patents relating to the content of the manuscript?
- Do you have any other financial competing interests?
- Do you have any non-financial competing interests in relation to this paper?

If you can answer no to all of the above, write 'I declare that I have no competing interests' below. If your reply is yes to any, please give details below.

I have no competing interests

I agree to the open peer review policy of the journal. I understand that my name will be included on my report to the authors and, if the manuscript is accepted for publication, my named report including any attachments I upload will be posted on the website along with the authors' responses. I agree for my report to be made available under an Open Access Creative Commons CC-BY license (http://creativecommons.org/licenses/by/4.0/). I understand that any comments which I do not wish to be included in my named report can be included as confidential comments to the editors, which will not be published.

Choose an item.

To further support our reviewers, we have joined with Publons, where you can gain additional credit to further highlight your hard work (see: https://publons.com/journal/530/gigascience). On publication of this paper, your review will be automatically added to Publons, you can then choose whether or not to claim your Publons credit. I understand this statement.

Yes Choose an item.
